# Supplementary material for: Distinctly different active sites of ZnO-ZrO2 catalysts in CO2 and CO hydrogenation to methanol reactions
Source: Nat Commun. 2025 May 18;16:4622. doi: 10.1038/s41467-025-59996-5 (PMC12086193; doi:10.1038/s41467-025-59996-5)
Supplement: Supplementary file 1 — Supplementary Information [file 41467_2025_59996_MOESM1_ESM.pdf]

Supporting Information

Distinctly different active sites of $ZnO\text{-}ZrO_2$ catalysts in CO_2 and CO hydrogenation to methanol reactions

Jieqiong Ding[1,2#], Yao Peng[3#], Wei Xiong[2], Dongdong Wang[2], Ziran Xu[4], Qinxue Nie[2], Zheng Jiang[4], Zhi-Pan Liu[3,5], Cheng Shang[3,5*], Weixin Huang[2*]

[1] Hefei National Research Center for Physical Sciences at the Microscale, University of Science and Technology of China, Hefei 230026, China

[2] State Key Laboratory of Precision and Intelligent Chemistry, iChEM, Key Laboratory of Surface and Interface Chemistry and Energy Catalysis of Anhui Higher Education Institutes, School of Chemistry and Materials Science, University of Science and Technology of China, Hefei 230026, China

[3] Collaborative Innovation Center of Chemistry for Energy Material, Shanghai Key Laboratory of Molecular Catalysis and Innovative Materials, Key Laboratory of Computational Physical Science, Department of Chemistry, Fudan University, Shanghai 200433, China

[4] National Synchrotron Radiation Laboratory, University of Science and Technology of China, Hefei, Anhui 230029, China

[5] Shanghai Qi Zhi Institute, Shanghai 200030, China

[#] These authors contribute equally.

* Corresponding Author: huangwx@ustc.edu.cn (WH) and cshang@fudan.edu.cn (CS)

The PDF file includes:

Supplementary Figure 1 to 72

Supplementary Table 1 to 9

Supplementary References

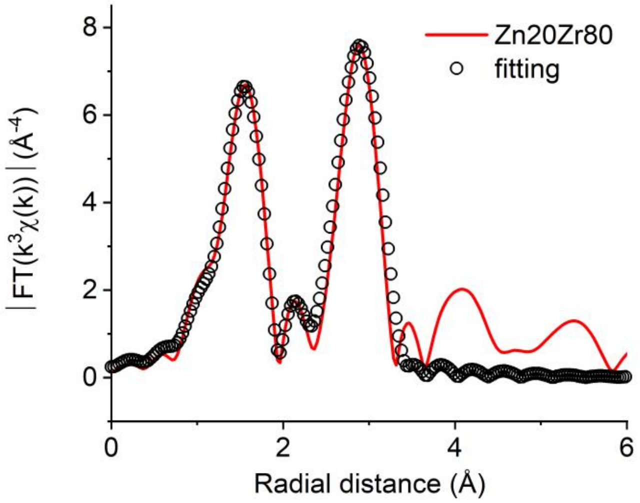

Supplementary Figure 1. Zn K-edge EXAFS fitting analyses for Zn20Zr80 samples in R Space.

Supplementary Table 1. EXAFS curve-fitting parameters for corresponding samples. [a]

Sample	Coordination	CN	R(Å)	$\sigma^2 \times 10^{-3}$(Å^2)	ΔE(eV)	R-factor
Zn foil	Zn-Zn	6	2.64	--	--	--
Zn20Zr80	Zn-O	4	1.98	7.04	1.2	1.90%
	Zn-O-Zn (Zr)	12.2	3.22	13.34	1.2	
ZnO	Zn-O	4	1.97			
	Zn-O-Zn	12	3.21			

[a] CN, coordination number; R, distance between absorber and backscatter atoms; σ^2, Debye-Waller factor to account for both thermal and structural disorders; ΔE, inner potential correction; R-factor indicates the goodness of the fit.

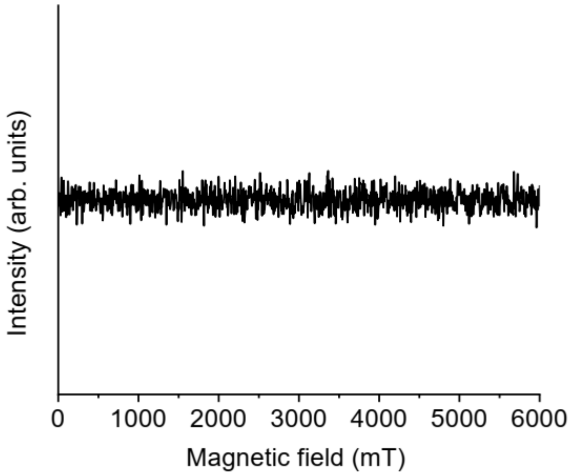

Supplementary Figure 2. ESR spectrum of Zn20Zr80 calcined in Ar at 773 K without exposure to air measured within the 0 to 6000 G range at 130 K. The adopted JEOL JES-FA200 ESR spectrometer outputs a fixed 8192 data points no matter how wide the test ranges are, thus a wide range ESR measurement of the 0 to 6000 G range is not good at characterizing the paramagnetic species with narrow ESR signals such as the O_2^-, F^+ centers, Zr^{3+} and interstitial Zn_i^+ defects in our case (Figure 1d), but good at probing the transition metal paramagnetic ions with *d*-orbitals which have strong coupling effects. Thus, the wide-range ESR spectrum shown here suggests that our Zn20Zr80 sample does not have transition metal impurities.

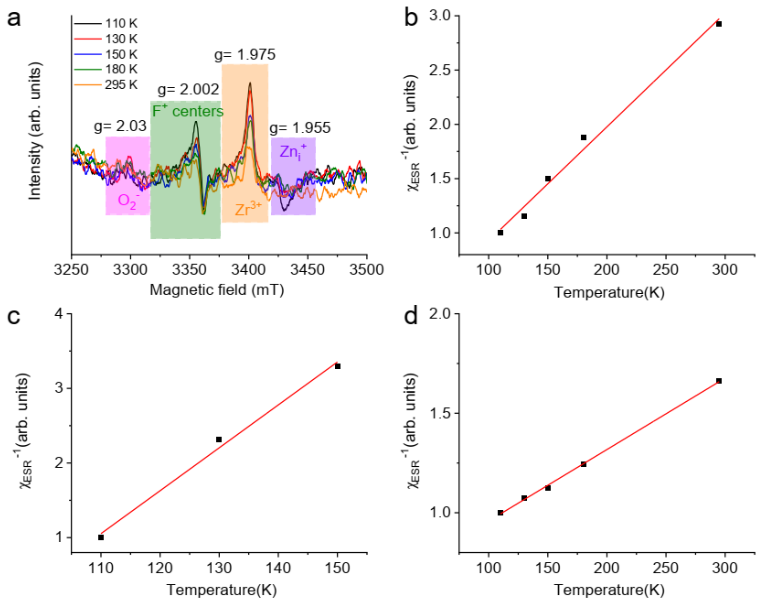

Supplementary Figure 3. a, ESR spectra of Zn20Zr80 calcined in Ar at 773 K without exposure to air measured at different temperature at the constant magnetic field modulation (3.500 G) and radio frequency power (2.000mW). Temperature dependence of the inverse EPR susceptibility χ_{EPR}^{-1} of the three observed ESR signals of **b**, F^+ centers, **c**, Zn_i^+ and **d**, Zr^{3+}. The ESR signals decrease with the measuring temperature of ESR spectra increasing. The O_2^- and Zn_i^+ ESR signals could not be detected when the measurement temperatures rose to 150 and 180 K, respectively. The plots of χ_{EPR} of observed F^+ centers, Zr^{3+}, and Zn_i^+ ESR signals against the measurement temperatures follow the Curie-Weiss law, confirming that the observed ESR signals arise from the pragmatically isolated sites. However, the plot of χ_{EPR} of observed O_2^- ESR signals against the measurement temperatures could not be made because it had only two data.

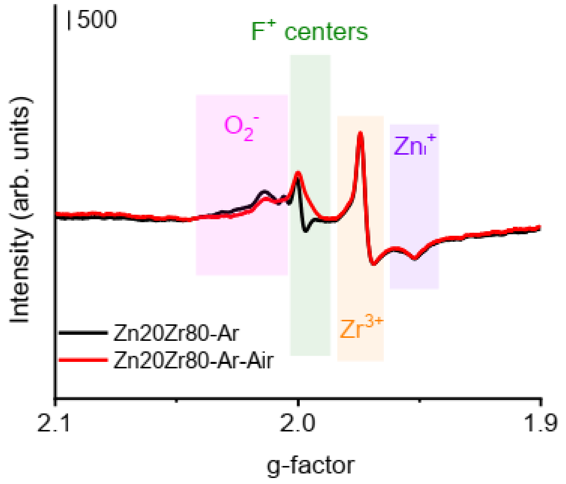

Supplementary Figure 4. ESR spectra of Zn20Zr80 calcined in Ar at 773 K without exposure to air (black), and then exposed to air for 10 min (red).

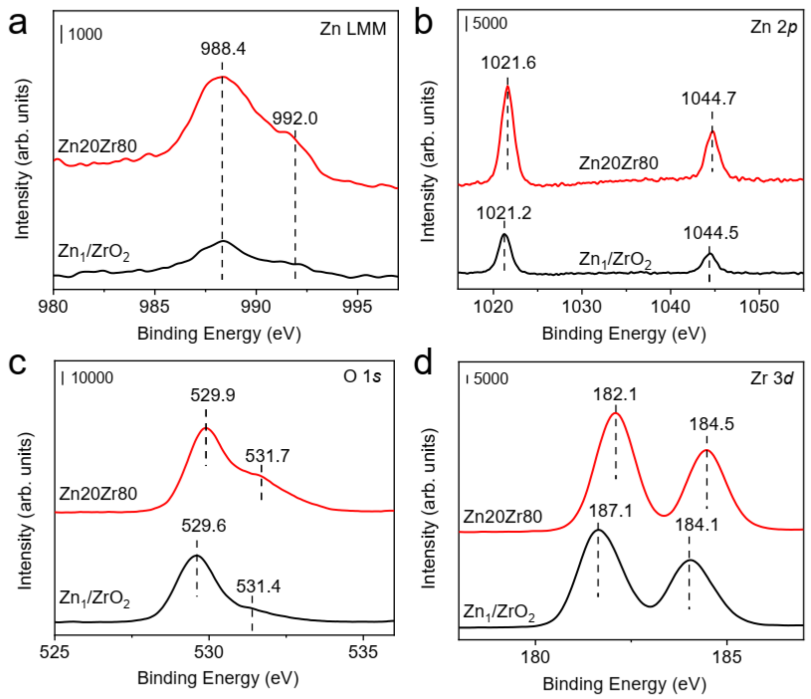

Supplementary Figure 5. a to **d,** Zn LMM, Zn 2*p*, O 1*s* and Zr 3*d* X-ray Photoelectron Spectroscopy spectra of Zn20Zr80. Zn LMM Auger spectra with the maximum at 988.4 eV and shoulder at ~992 eV (kinetic energy) can be clearly associated with Zn^{2+}. No metallic Zn is observed [1,2]. The Zn 2*p*3/2 peaks at binding energies of 1021.6 and 1021.2 eV can attribute to Zn^{2+} with different coordination structures [3]. The Zr $3d_{5/2}$ peaks at 181.7 and 182.1 eV can be assigned to Zr^{4+} cations in *t*-ZrO_2 and Zn^{2+}-substituted ZrO_2 solid solutions [4, 5]. The O 1*s* spectra show the main peak at 529.6 to 529.9 eV related to lattice oxygen with the broad shoulder peak at 531.4 to 531.7 eV associated with both surface hydroxyl and surface carbonate species.

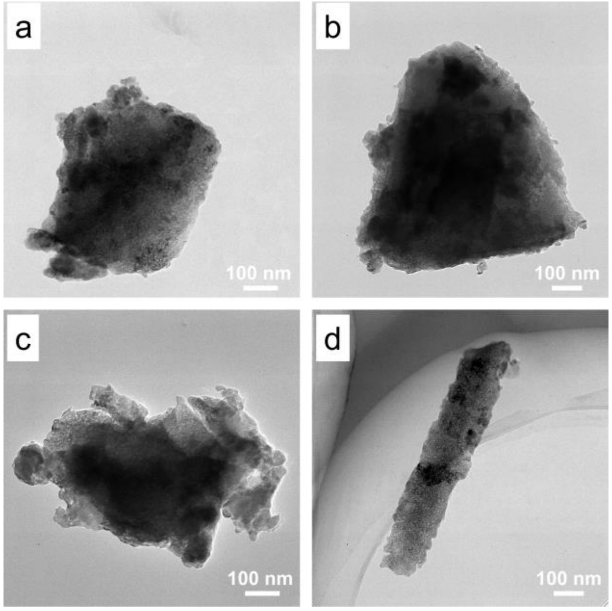

Supplementary Figure 6. a to **d**, TEM image of the Zn20Zr80 catalyst.

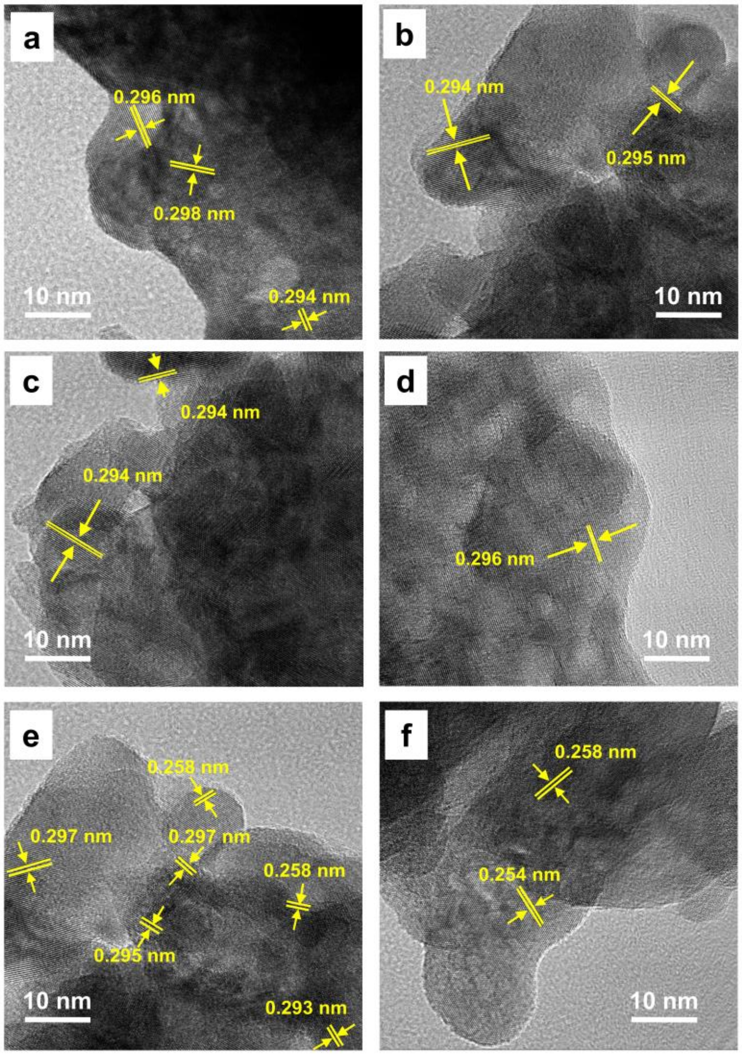

Supplementary Figure 7. a to **f**, HRTEM image of the Zn20Zr80 catalyst, 0.258 nm and 0.296 nm double lines correspond to the interplane spacings of (002) and (101) planes of planes of *t*-ZrO_2, respectively.

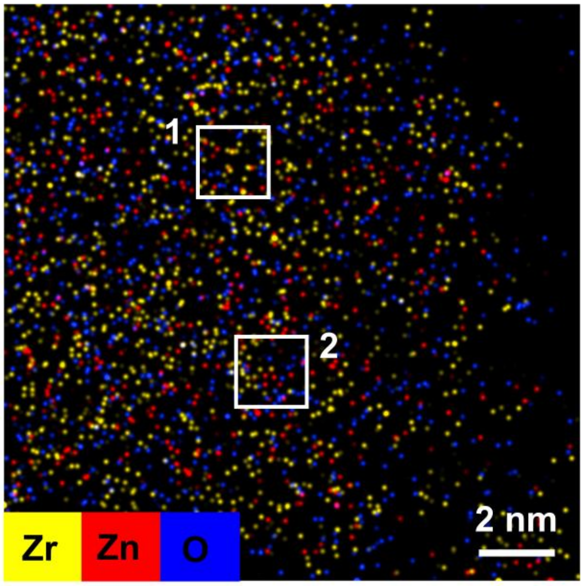

Supplementary Figure 8. EDS mapping image of the Zn20Zr80 catalyst correspond with atomically resolved STEM images in Fig. 1g.

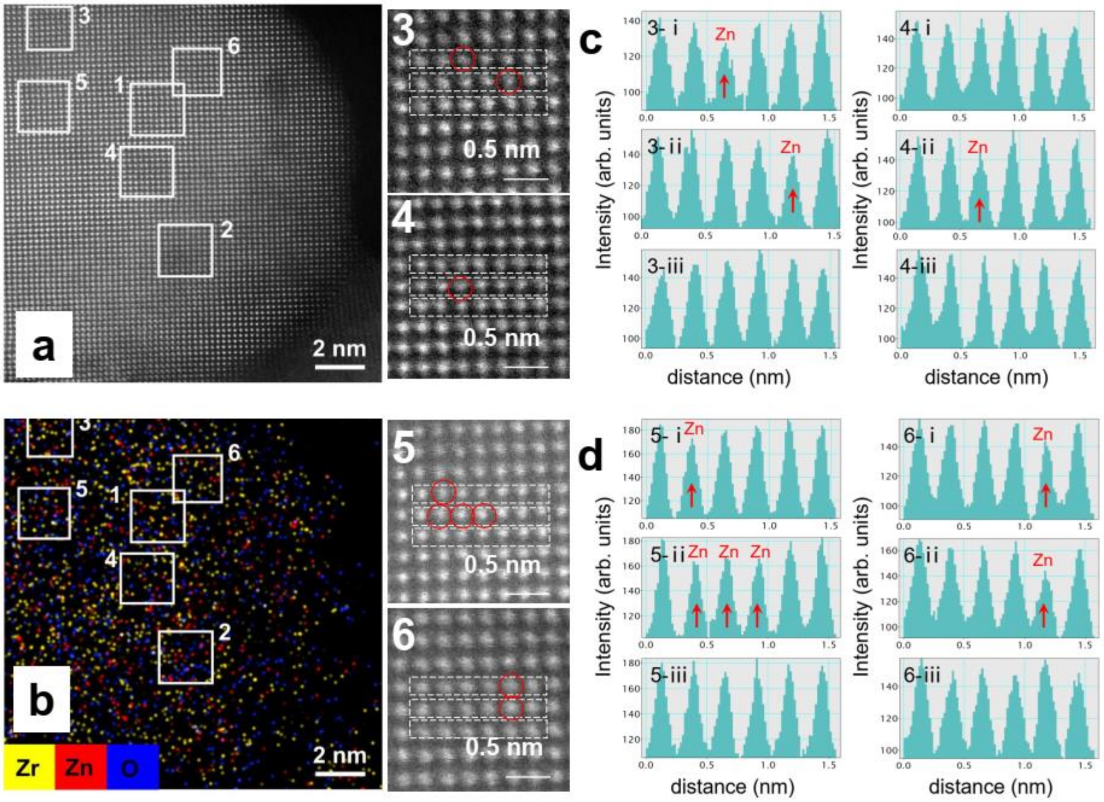

Supplementary Figure 9. a, atomically resolved STEM spectrum imaging the edge of the nanoparticle. **b**, EDS mapping images of the Zn20Zr80 catalyst. **c** to **d**, the corresponding single-pixel line profiles across the atom rows marked by rectangles in panel 3-i, 3-ii, 3-iii; 4-i, 4-ii, 4-iii; 5-i, 5-ii, 5-iii; 6-i, 6-ii, 6-iii. The dark dots and low-intensity single-pixel line profiles correspond to Zn-containing atomic columns, marked with red circles and arrows.

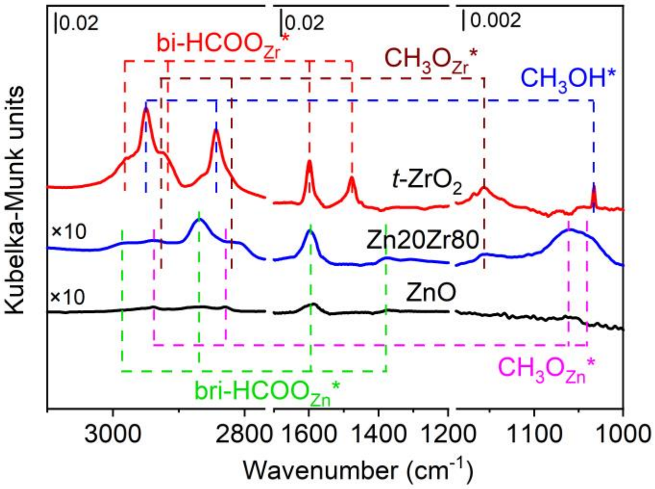

Supplementary Figure 10. In-situ spectra of CH_3OH adsorption at 303 K of *t*-ZrO_2, ZnO and Zn20Zr80.

Supplementary Table 2. Catalytic performance of Zn20Zr80 catalyst in CO_2 hydrogenation reaction under 3 MPa CO_2+H_2 (H_2: CO_2 = 3; flow rate: 30 mL min^{-1}; catalyst mass: 600 mg Zn20Zr80 diluted with 400 mg SiC)

Temperature (K)	CO_2 Conversion (%)	Selectivity (%)			
		CH_3OH	CO	CH_4	C_{2+}[0]
523	1.07	89.8	8.1	1.2	0.4
533	1.59	88.2	10.7	0.4	0.3
548	2.89	88.0	11.4	0.3	0.3

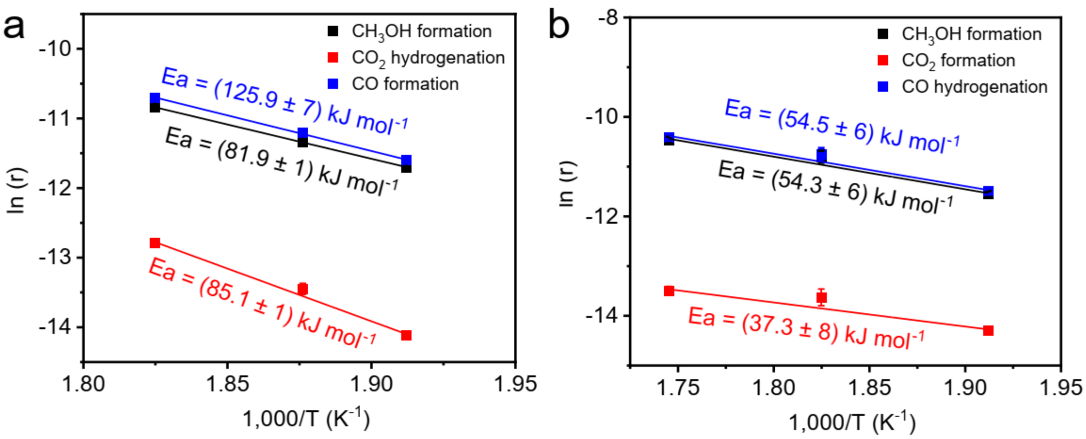

Supplementary Figure 11. Arrhenius plots of **a,** CO_2 hydrogenation, CO or CH_3OH formation and **b,** CO hydrogenation, CO_2 or CH_3OH formation of Zn20Zr80 catalyst derived from catalytic performance results in supplementary Table 2 and Figure 2c. The error bars in the figure represent the standard errors (SE) of the fitted values.

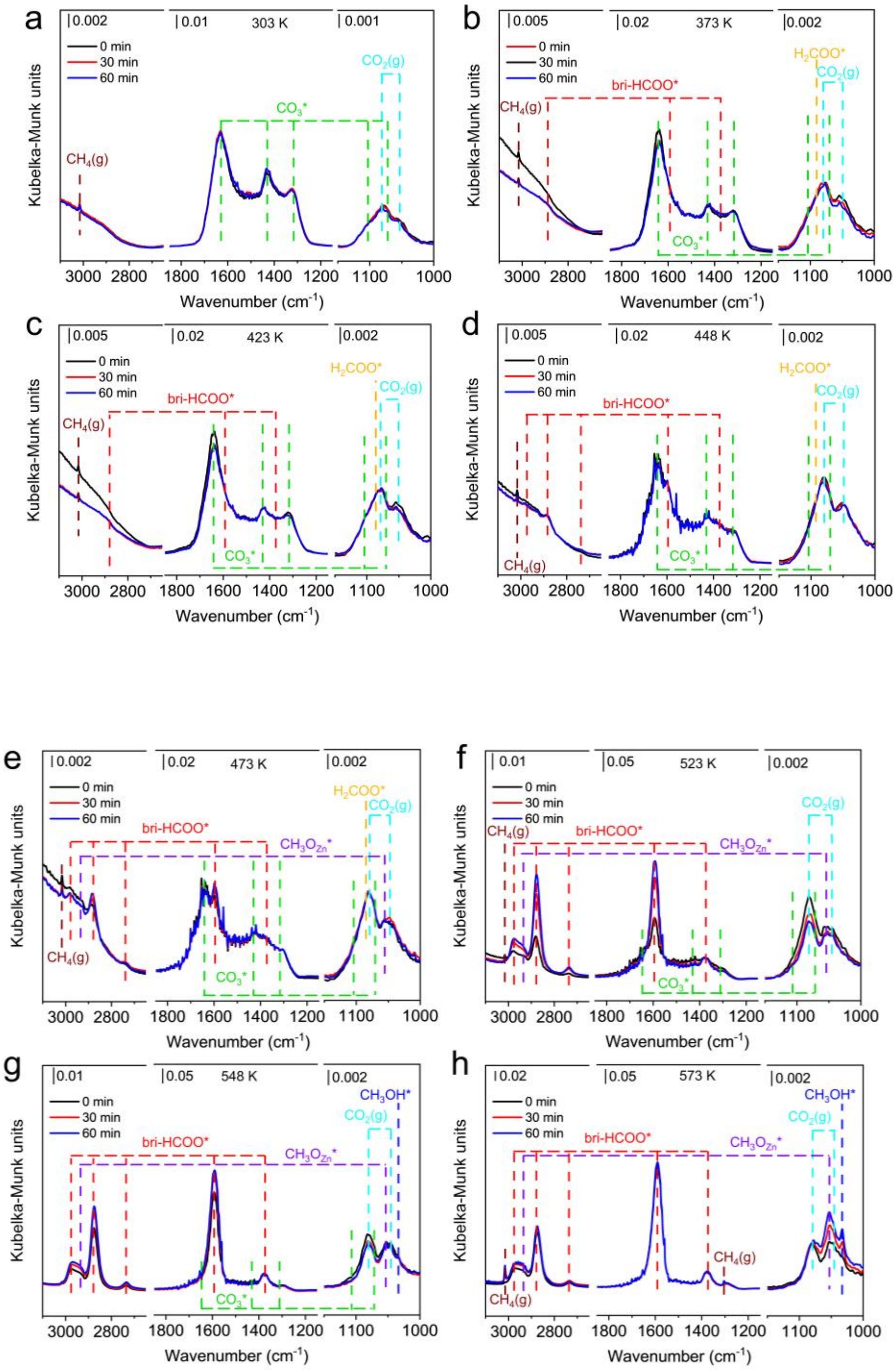

Supplementary Figure 12. Temporal in situ DRIFTS spectra of Zn20Zr80 exposed to 3 MPa CO_2+H_2 (H_2: CO_2 = 3) atmosphere at **a**, 303 K. **b**, 373 K. **c**, 423 K. **d**, 448 K. **e**, 473 K. **f**, 523 K. **g**, 548 K. **h**, 573 K.

Supplementary Table 3. Assignment of vibrational bands observed upon reaction on Zn20Zr80 and Zn_1/*m*-ZrO_2.

Species	Assignment	Peaks(cm^{-1})		Ref.
		DFT	Experiment	
Bridge Formates (bri-HCOO)	υ_{as}(OCO)	1563(bri-$HCOO_{Zn, Zr}$*),1556(bri-$HCOO_{Zr, Zr}$*)	1590-1597(bri-$HCOO_{Zn, Zr}$*/bri-HCOO*-1590),1565-1560(bri-HCOO*-1561)	6-9
	υ_{a}(OCO)	1363(bri-$HCOO_{Zn, Zr}$*),1361(bri-$HCOO_{Zr, Zr}$*)	1361-1370(bri-$HCOO_{Zn, Zr}$*/bri-HCOO*-1590),1450-1430(bri-HCOO*-1561)	
	υ (CH)		2868-2884	
	δ(CH)		1377-1383	
	δ(CH)+ υ_{as}(OCO)		2952-2973	
	δ(CH)+ υ_{s}(OCO)		2733-2740	
Methoxys (CH_3O)	υ (CH_3)		2922-2942	10-13
	υ (CH_3)		2810-2831	
	υ (OCH_3)	1037(CH_3O_{Zn}*) 1125(CH_3O_{Zr}*)	1042-1060(CH_3O_{Zn}*) 1143-1145(CH_3O_{Zr}*)	
dioxymethylenes (H_2COO)	υ_{s}(OCO)	1074	1072-1089	14-18
	υ (CH_2)		2910-2912	
Protonated formic acid (H_2COOH)	υ(OCO)	1160	1197-1202	18
Carbonates (CO_3^{2-})	υ(OCO)		1650-1620,1504-1550,1370-1490,1297-1318,1092-1107	18, 19
Carbon dioxide (CO_2)	υ(OCO)		2324-2342	20-23
Methanol (CH_3OH)	υ(OCO)		1032	24
	υ (CH)		2952,2903	
Hydroxyl group (OH)	υ (OH)		3648-3690,3720-3770	25, 26
Water (H_2O)	υ (H_2O)		3600	24
	δ(H_2O)		1632	
Carbon dioxide gas (CO_2)			3750-3500,2200-2400,1050,1076	27
Methane gas (CH_4)			3015,1305	27

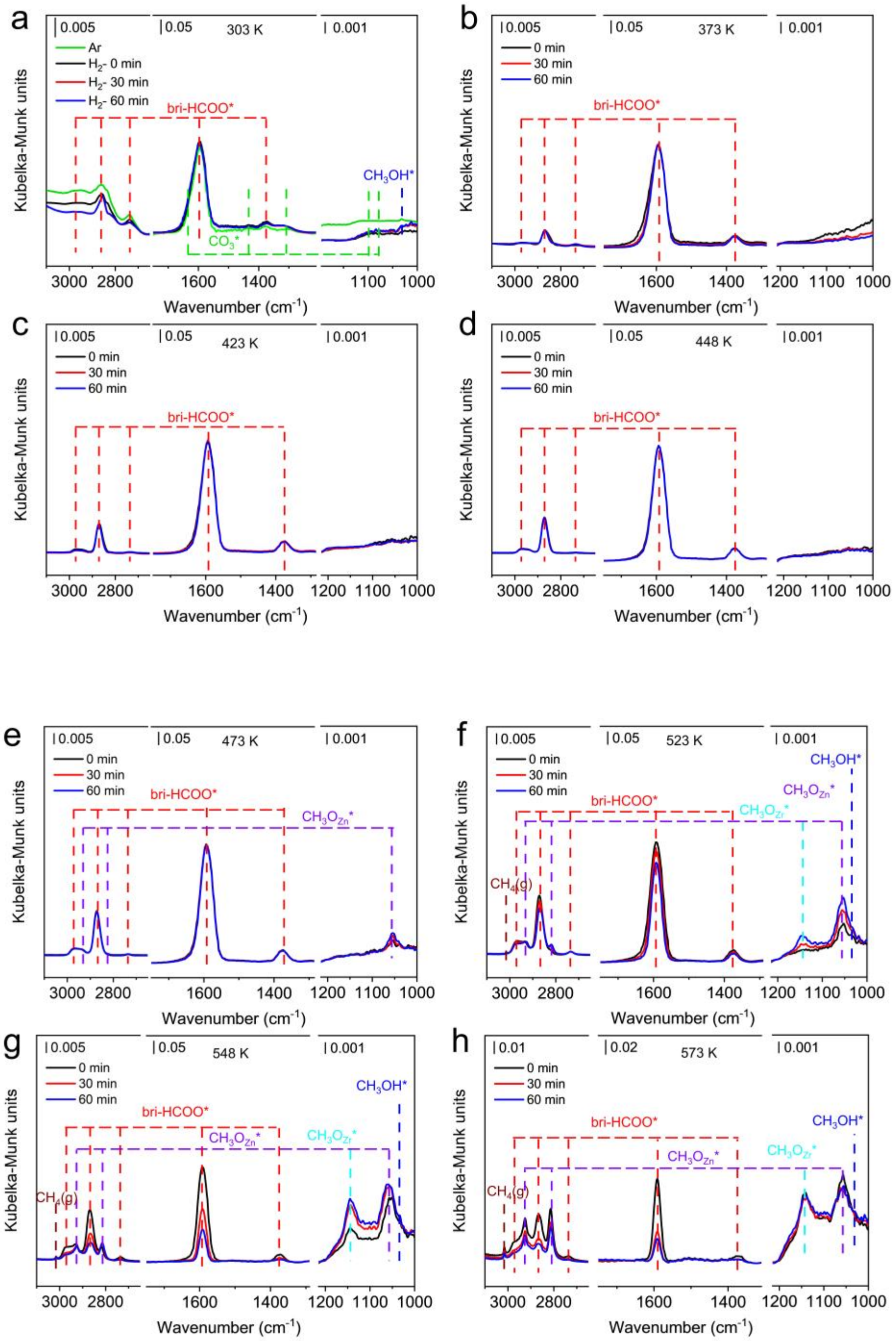

Supplementary Figure 13. Temporal in situ DRIFTS spectra of Zn20Zr80 exposed to 3 Mpa H_2 (pretreated in 3 MPa CO_2+H_2 (H_2: CO_2 = 3) at 573 K for 60 min, then purged in Ar) at **a**, 303 K. **b**, 373 K. **c**, 423 K. **d**, 448 K. **e**, 473 K. **f**, 523 K. **g**, 548 K. **h**, 573 K.

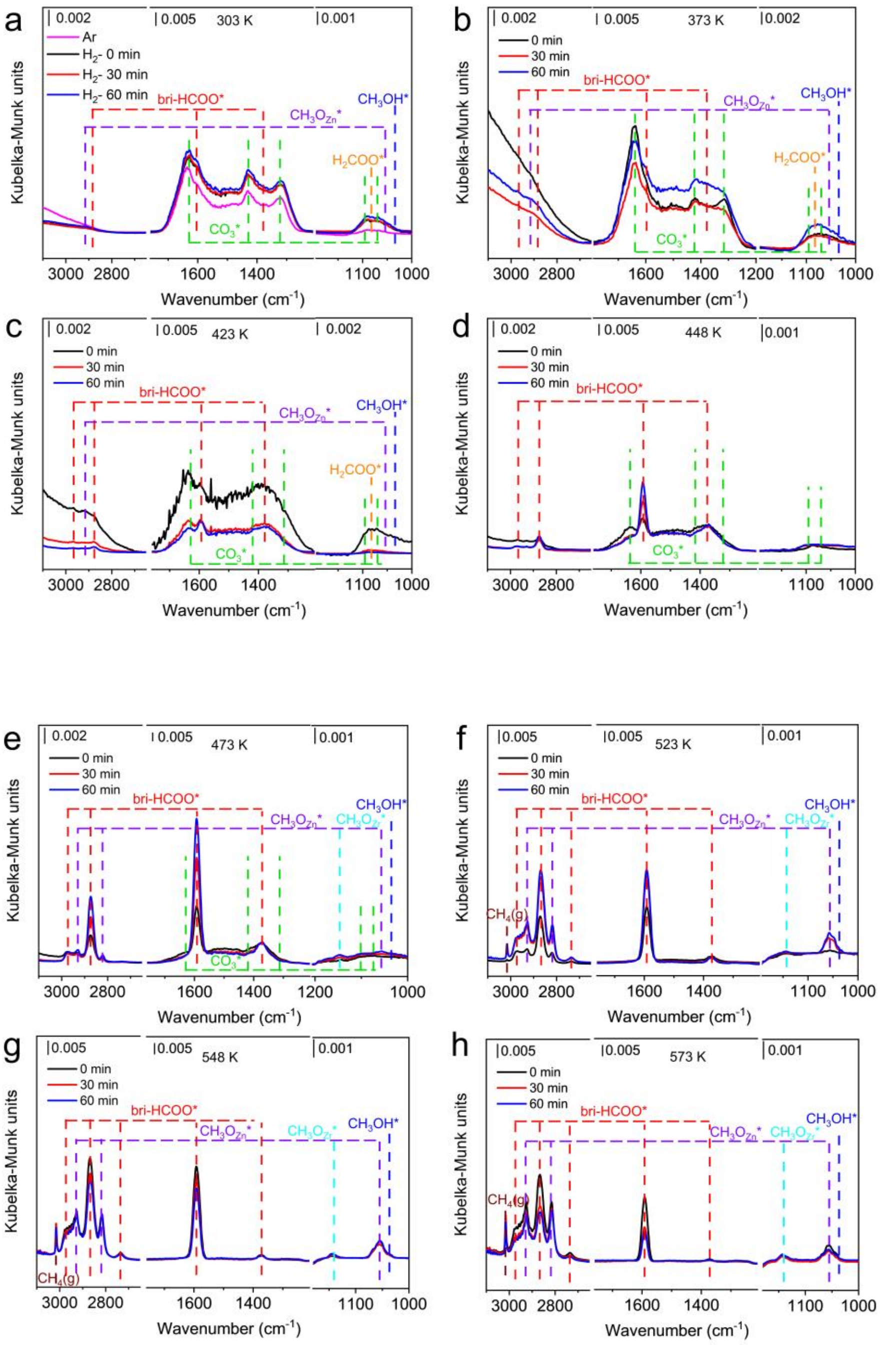

Supplementary Figure 14. Temporal in situ DRIFTS spectra of Zn20Zr80 exposed to 3 Mpa H_2 (pretreated in 3 MPa CO_2 at 303 K for 60 min, then purged in Ar) at **a**, 303 K. **b**, 373 K. **c**, 423 K. **d**, 448 K. **e**, 473 K. **f**, 523 K. **g**, 548 K. **h**, 573 K.

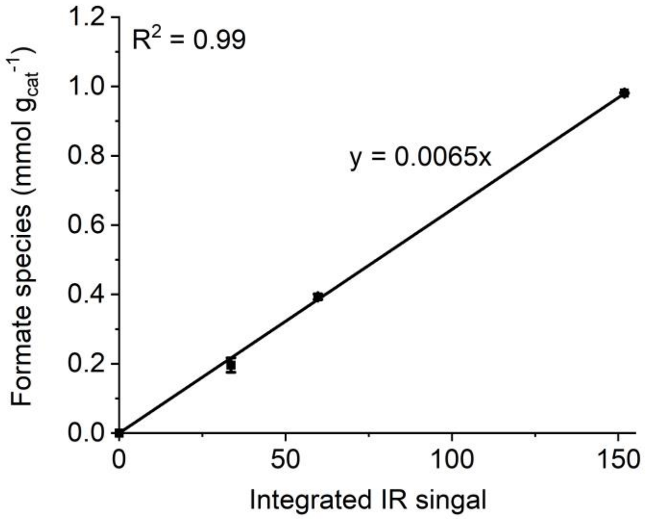

Supplementary Figure 15. Calibration curves of IR signal for formate species over Zn20Zr80. The error bars in the figure represent the standard errors (SE) of the fitted values.

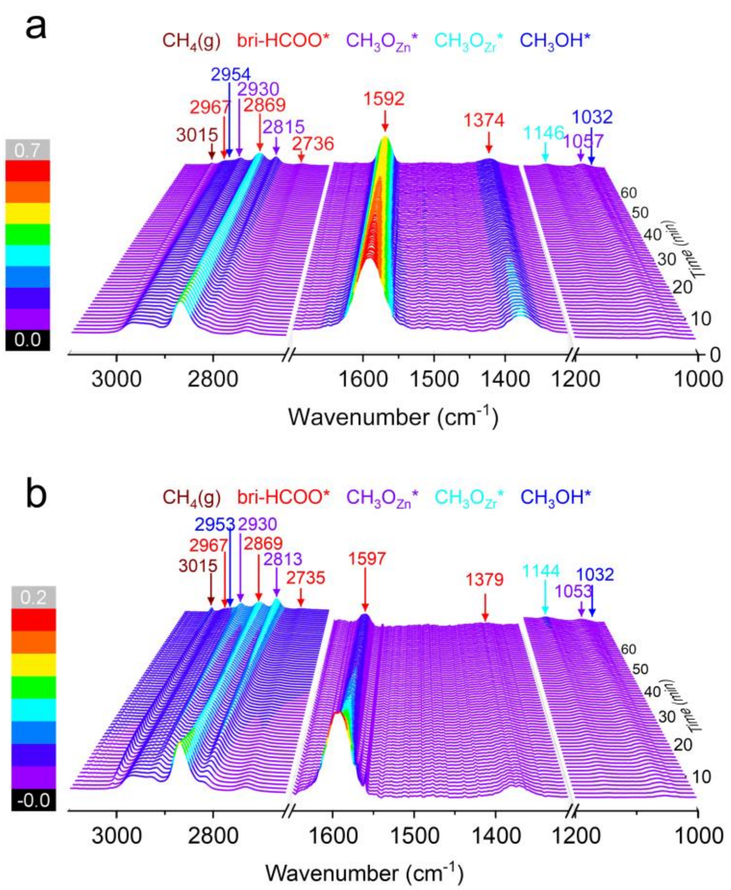

Supplementary Figure 16. Temporal in situ DRIFTS spectra of Zn20Zr80 (pretreated in 3 MPa CO_2+H_2 (H_2: CO_2 = 3) at 573 K for 60 min, then purged in Ar) exposed to 3 MPa H_2 at **a**, 548 K and **b**, 573 K.

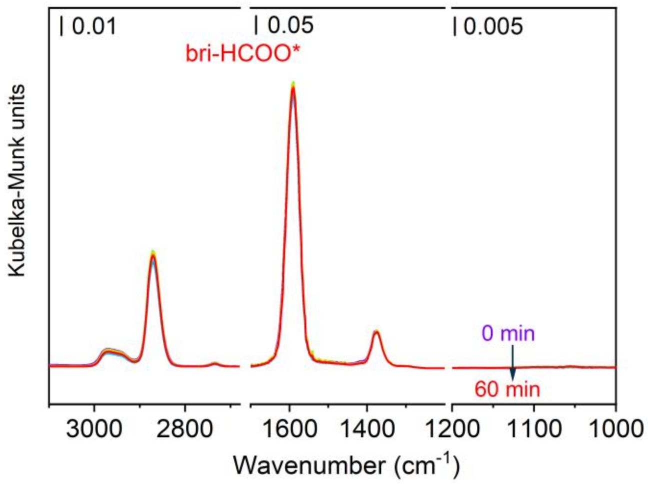

Supplementary Figure 17. Temporal in situ DRIFTS spectra of Zn20Zr80 exposed to 3 MPa H_2 (pretreated in 3 MPa CO_2+H_2 (H_2: CO_2 = 3) at 573 K for 60 min, then purged in Ar) at 463 K.

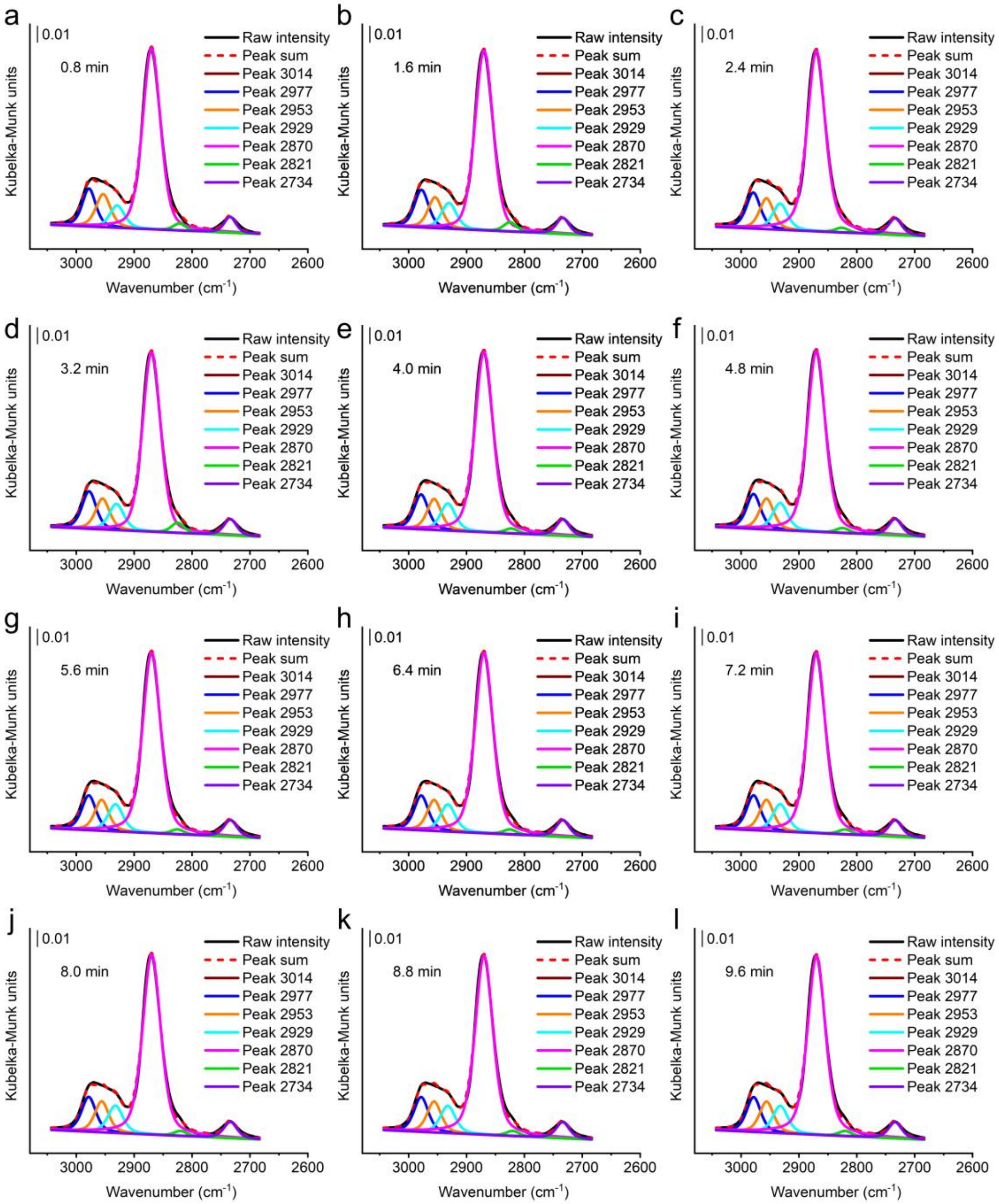

Supplementary Figure 18. a to **l,** peak-fitted time-resolved in situ DRIFTS spectra of Zn20Zr80 exposed to 3 MPa H_2 (pretreated in 3 MPa CO_2+H_2 (H_2: CO_2 = 3) at 573 K for 60 min, then purged in Ar) at 523 K.

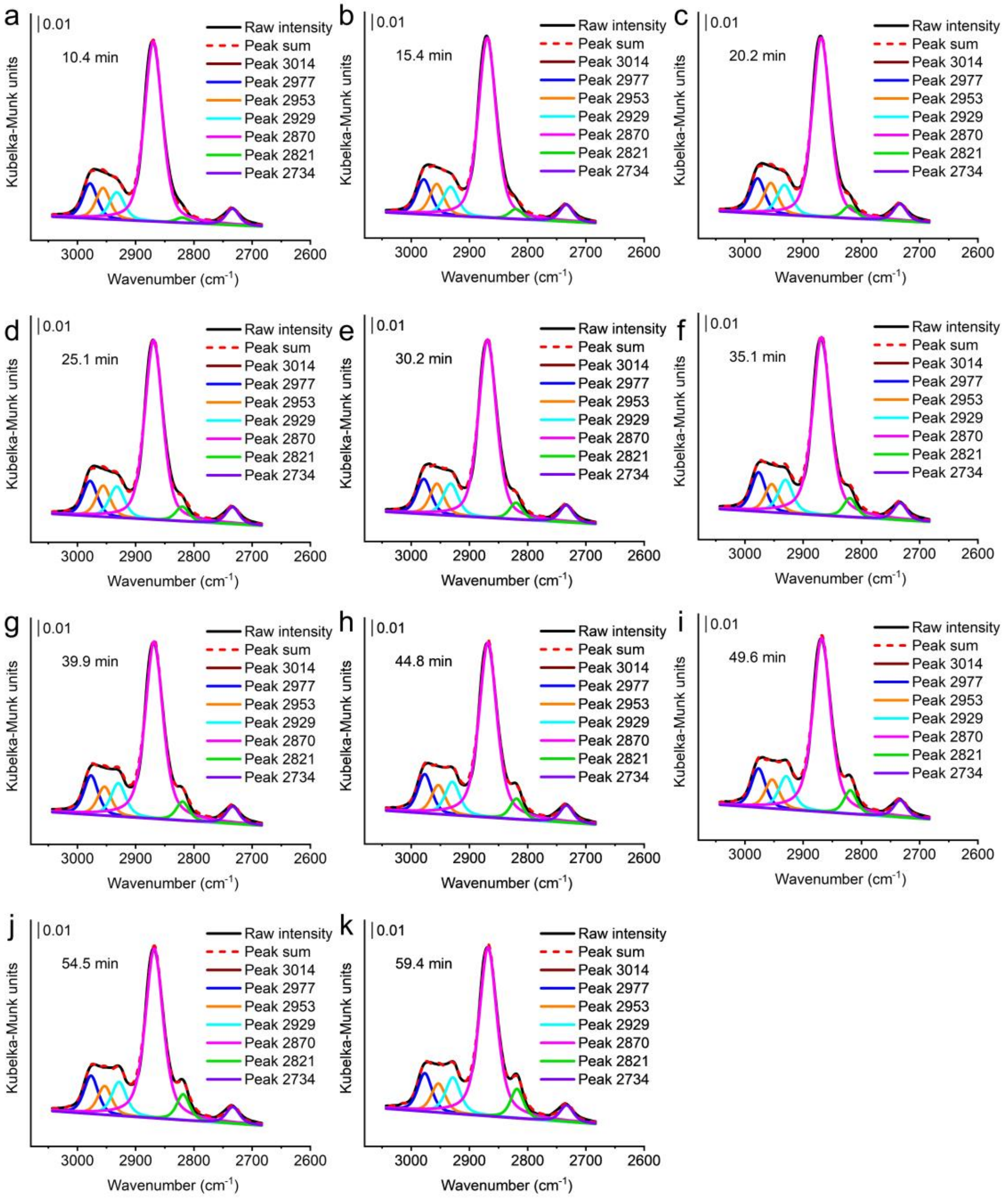

Supplementary Figure 19. a to **k,** peak-fitted time-resolved in situ DRIFTS spectra of Zn20Zr80 exposed to 3 MPa H_2 (pretreated in 3 MPa CO_2+H_2 (H_2: CO_2 = 3) at 573 K for 60 min, then purged in Ar) at 523 K.

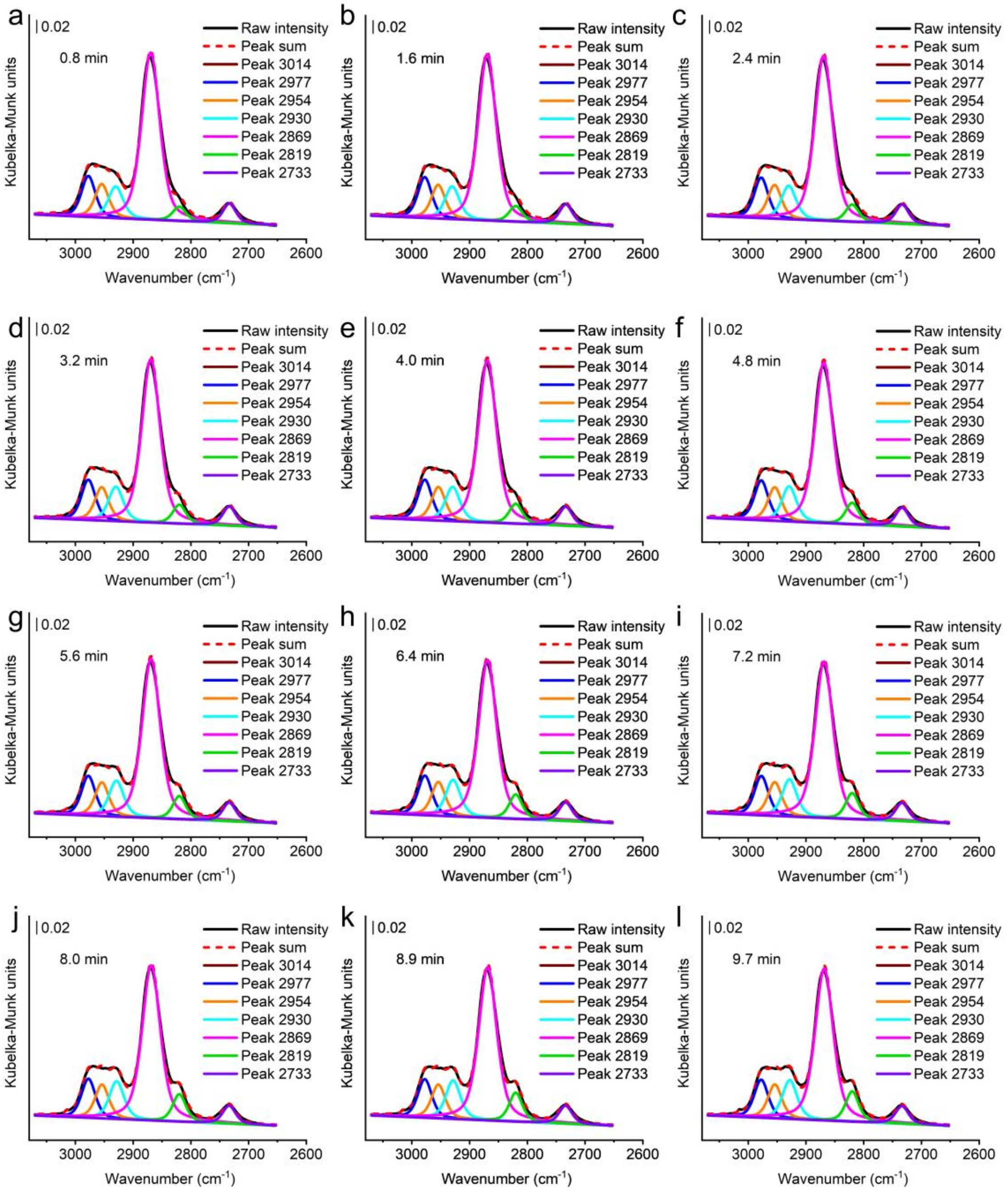

Supplementary Figure 20. a to **l,** peak-fitted time-resolved in situ DRIFTS spectra of Zn20Zr80 exposed to 3 MPa H_2 (pretreated in 3 MPa CO_2+H_2 (H_2: CO_2 = 3) at 573 K for 60 min, then purged in Ar) at 548 K.

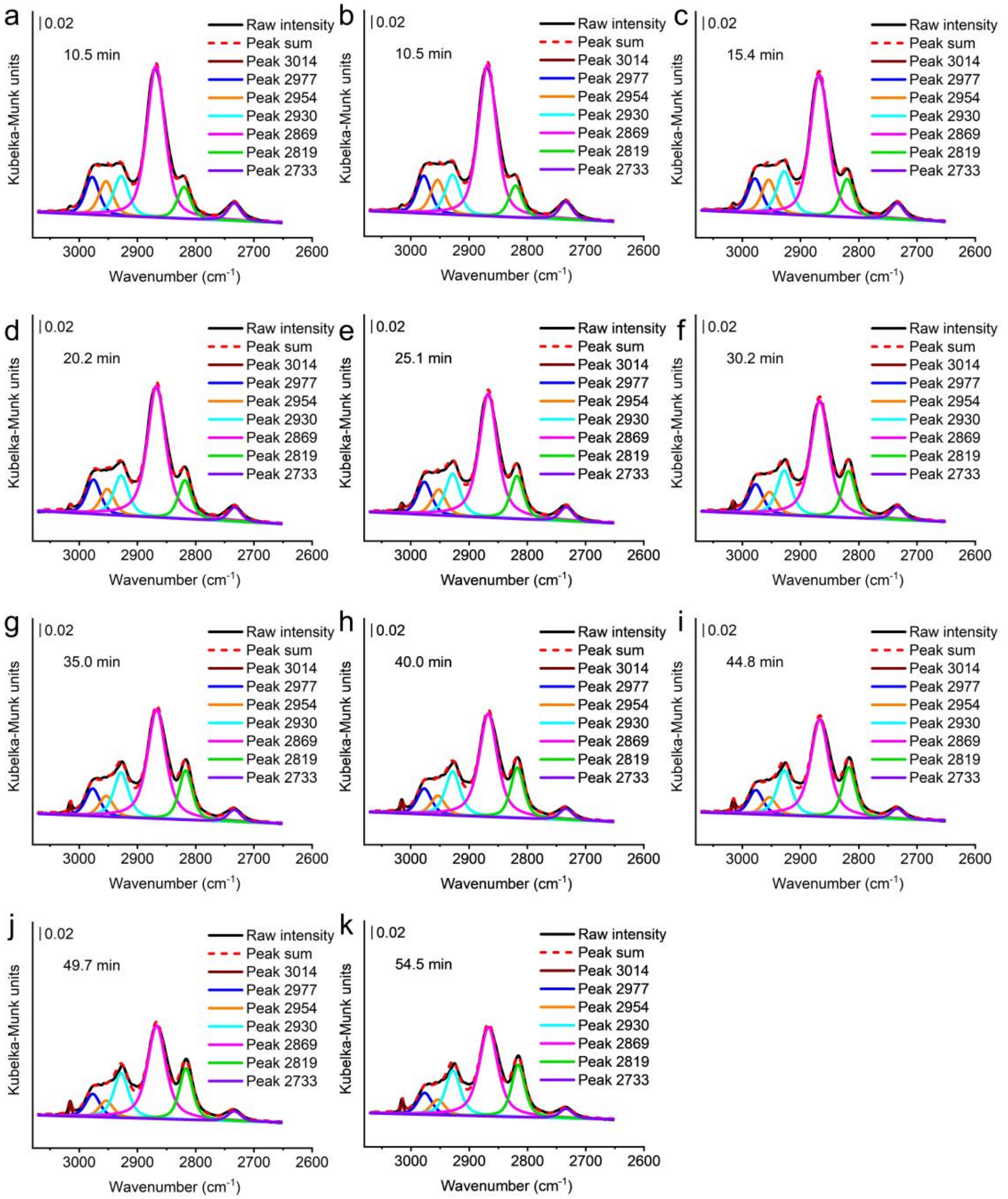

Supplementary Figure 21. a to **k,** peak-fitted time-resolved in situ DRIFTS spectra of Zn20Zr80 exposed to 3 MPa H_2 (pretreated in 3 MPa CO_2+H_2 (H_2: CO_2 = 3) at 573 K for 60 min, then purged in Ar) at 548 K.

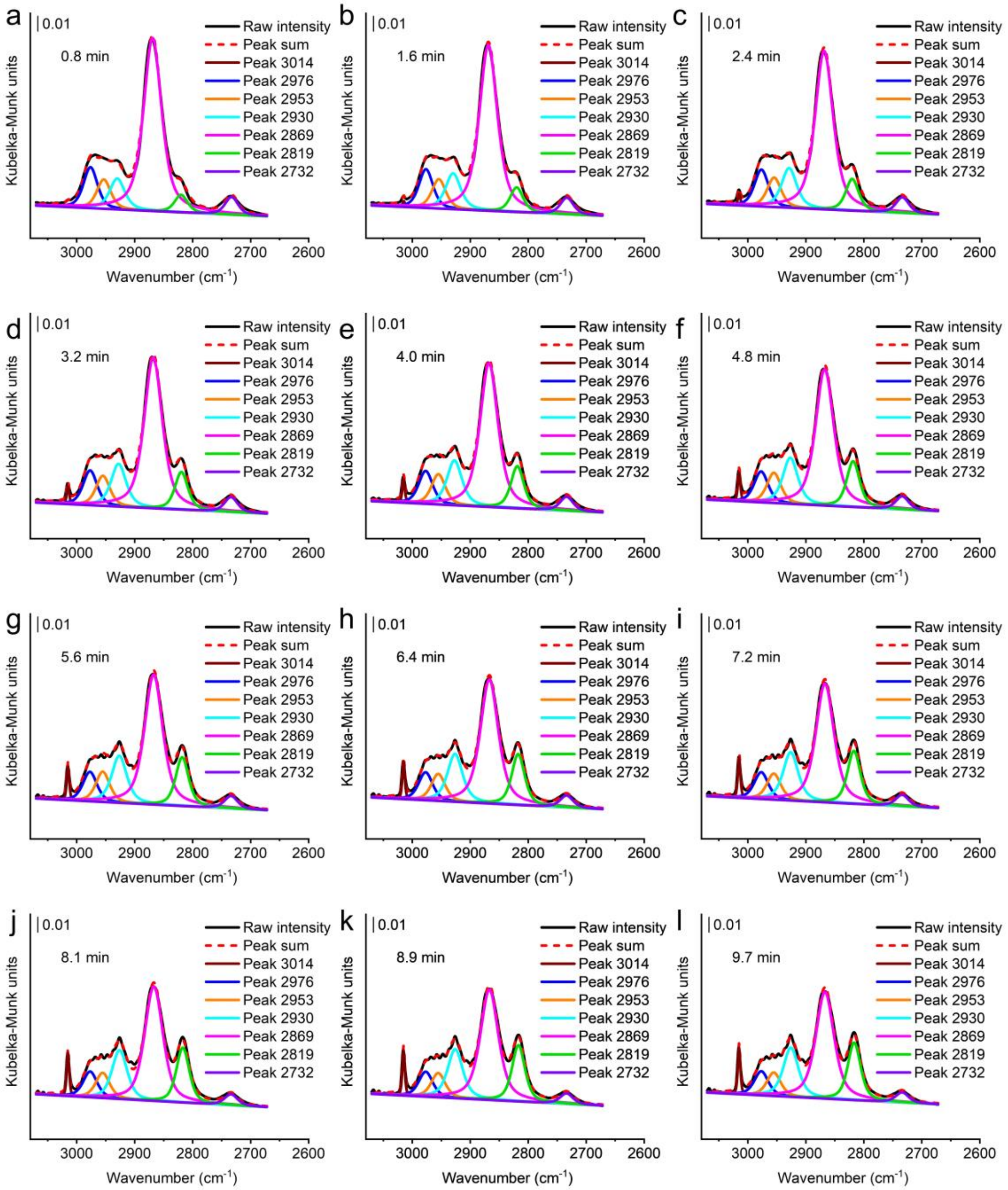

Supplementary Figure 22. a to **l,** peak-fitted time-resolved in situ DRIFTS spectra of Zn20Zr80 exposed to 3 MPa H_2 (pretreated in 3 MPa CO_2+H_2 (H_2: CO_2 = 3) at 573 K for 60 min, then purged in Ar) at 573 K.

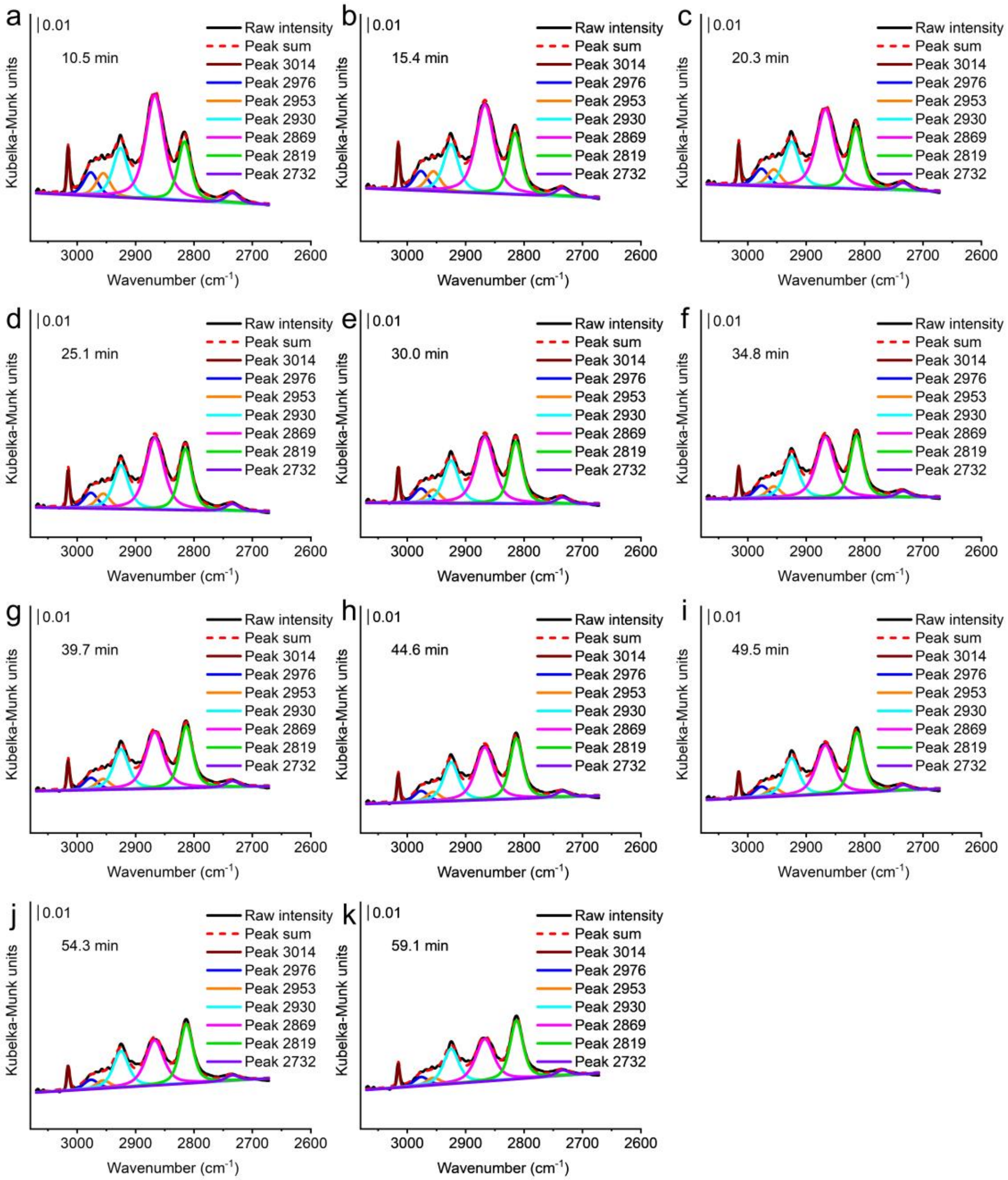

Supplementary Figure 23. a to **k,** peak-fitted time-resolved in situ DRIFTS spectra of Zn20Zr80 exposed to 3 MPa H_2 (pretreated in 3 MPa CO_2+H_2 (H_2: CO_2 = 3) at 573 K for 60 min, then purged in Ar) at 573 K.

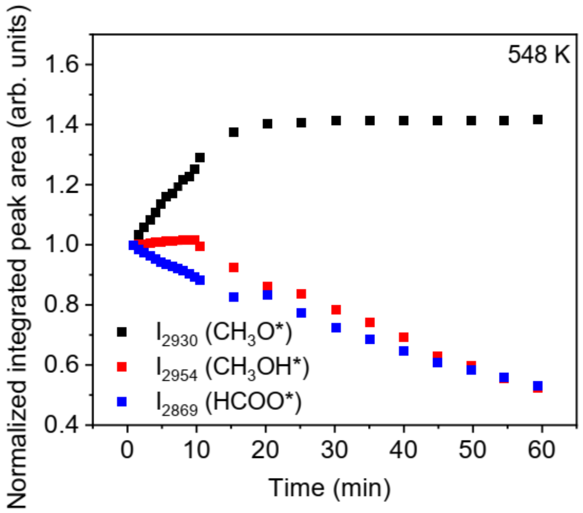

Supplementary Figure 24. The corresponding normalized integrated peak area of observed species in Supplementary Figure 16a as a function of time on Zn20Zr80 catalyst at 548 K.

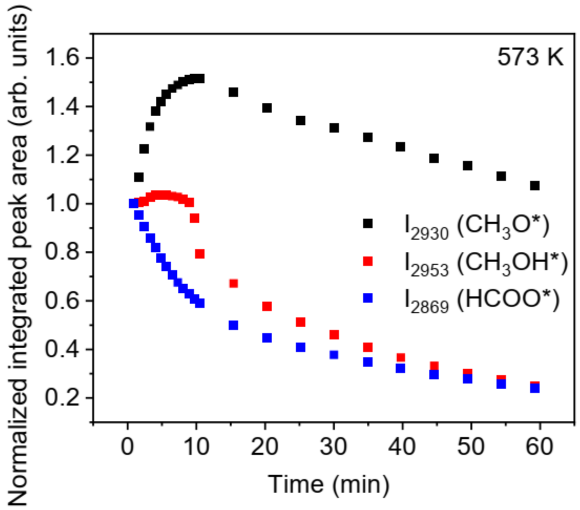

Supplementary Figure 25. The corresponding normalized integrated peak area of observed species in Supplementary Figure 16b as a function of time on Zn20Zr80 catalyst at 573 K.

$$HCOO* + 2H* \xrightarrow{r_1} CH_3O*$$ rate-limiting step

$$CH_3O* + H* \xrightarrow{r_2} CH_3OH*$$ in equilibrium

$$CH_3OH* \xrightarrow{r_{-2}} CH_3O* + H*$$

$$CH_3OH* \xrightarrow{r_3} CH_3OH(g)$$ in equilibrium

$$CH_3OH(g) \xrightarrow{r_{-3}} CH_3OH*$$

Supplementary Figure 26. Elementary surface reaction kinetic of high-temperature surface reaction pathway starting from bri-HCOO* with the bri-HCOO* hydrogenation reaction as the rate-limiting step.

$$r_{HCOO*} = -\frac{d[HCOO*]}{dt} = r_1 = k_1 \times [H*]^2 \times [HCOO*]$$

During the hydrogenation of bri-HCOO* in 3 MPa H_2, the [H*] can be considered as a constant. Thus,

$$r_{HCOO*} = -\frac{d[HCOO*]}{dt} = k_{1,app} \times [HCOO*]$$

Assuming that the relative surface coverage of HCOO* is proportional to the area of its IR vibrational peaks at 2869 and 1590 cm^{-1},

$$-\frac{d[I_{2869\ cm^{-1}}]}{dt} = k_{1,app,2869} \times [I_{2869\ cm^{-1}}]\text{ , then } \ln\frac{[I_{2869\ cm^{-1}}]_t}{[I_{2869\ cm^{-1}}]_0} = k_{1,app,2869} \times t$$

$$-\frac{d[I_{1590\ cm^{-1}}]}{dt} = k_{1,app,1590} \times [I_{2869\ cm^{-1}}]\text{ , then } \ln\frac{[I_{1590\ cm^{-1}}]_t}{[I_{1590\ cm^{-1}}]_0} = k_{1,app,1590} \times t$$

Thus, $k_{1,app,2869}$ and $k_{1,app,1590}$ are the slopes of the fitted lines of $\ln\frac{[I_{2869\ cm^{-1}}]_t}{[I_{2869\ cm^{-1}}]_0}$ versus *t* and of $\ln\frac{[I_{1590\ cm^{-1}}]_t}{[I_{1590\ cm^{-1}}]_0}$) versus *t* as shown in Figure 4 and Supplementary Figure 28. Then, the activation energy of HCOO* hydrogenation reaction can be calculated using either $k_{1,app,2869}$ or $k_{1,app,1590}$ data at different reaction temperatures.

Meanwhile,

$$r_{CH_3O*,app} = -\frac{d[CH_3O*]}{dt} = r_1 - r_2 + r_{-2}$$

From our experimental data (Figure 4b and Supplementary Figure 24 to 25), the CH_3OH^* coverages remain unchanged during the initial HCOO* hydrogenation reaction, suggesting that the CH_3OH^* is in equilibrium with CH_3O^* and gaseous CH_3OH ($r_2 = r_{-2}$; $r_3 = r_{-3}$)

Thus, during the initial HCOO* hydrogenation reaction,

$$r_{CH_3O*,app} = -\frac{d[CH_3O\ *]}{dt} = r_1$$

Assuming that the relative surface coverage of CH_3O^* is proportional to the area of its IR vibrational peak at 2930 cm^{-1}, then,

$$-\frac{d[I_{2930\ cm^{-1}}]}{dt} = r_{1,app}$$

Thus, the $r_{1,app}$ can be derived from the data of $[I_{2930\ cm^{-1}}]_t$ versus *t* during the initial HCOO* hydrogenation reaction (Supplementary Figure 27), and the activation energy of CH_3O^* formation reaction can be calculated using $r_{1,app}$ data at different reaction temperatures.

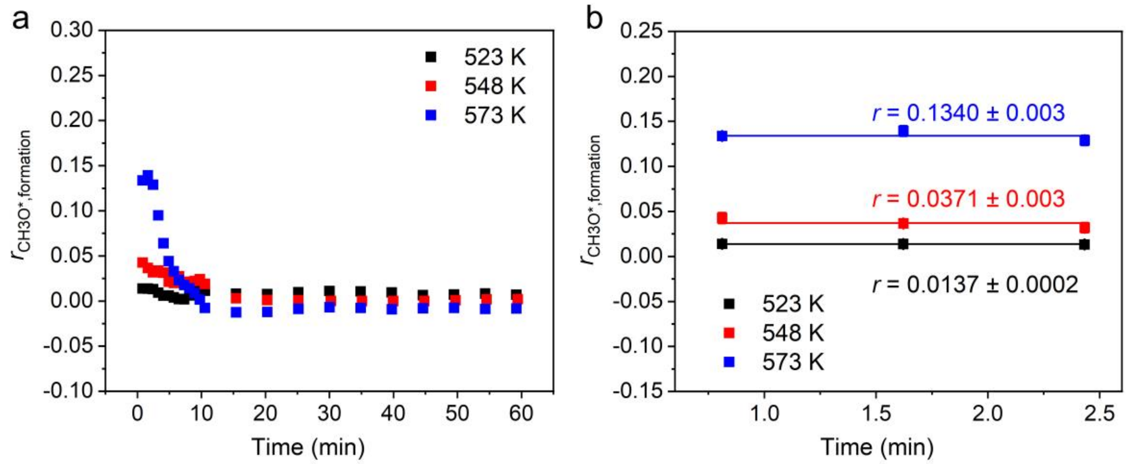

Supplementary Figure 27. a, the rate of CH_3O^* formation reaction on Zn20Zr80 at 523 K, 548 K and 573 K as a function of time derived from Fig. 4b, as well as Supplementary Figure 24 to 25. **b**, the average rate of CH_3O^* formation reaction on Zn20Zr80 at 0.8 to 2.5 min derived from panel (a). The error bars in the figure represent the standard errors (SE) of the fitted values.

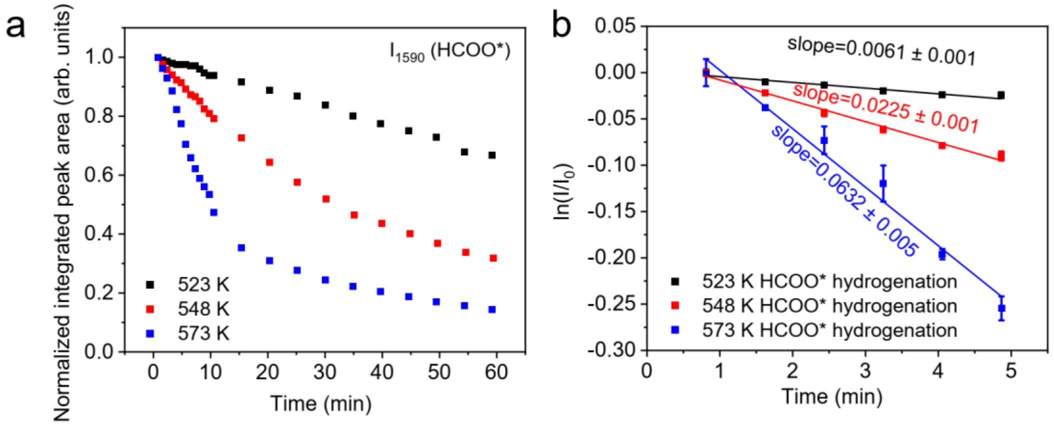

Supplementary Figure 28. a, the normalized integrated peak area of bri-HCOO* species in Figure 4a and Supplementary Figure 16 as a function of time on Zn20Zr80 catalyst. **b**, first-order reaction kinetic of bri-HCOO* hydrogenation reaction on Zn20Zr80 at different temperatures derived from panel (a). The error bars in the figure represent the standard errors (SE) of the fitted values.

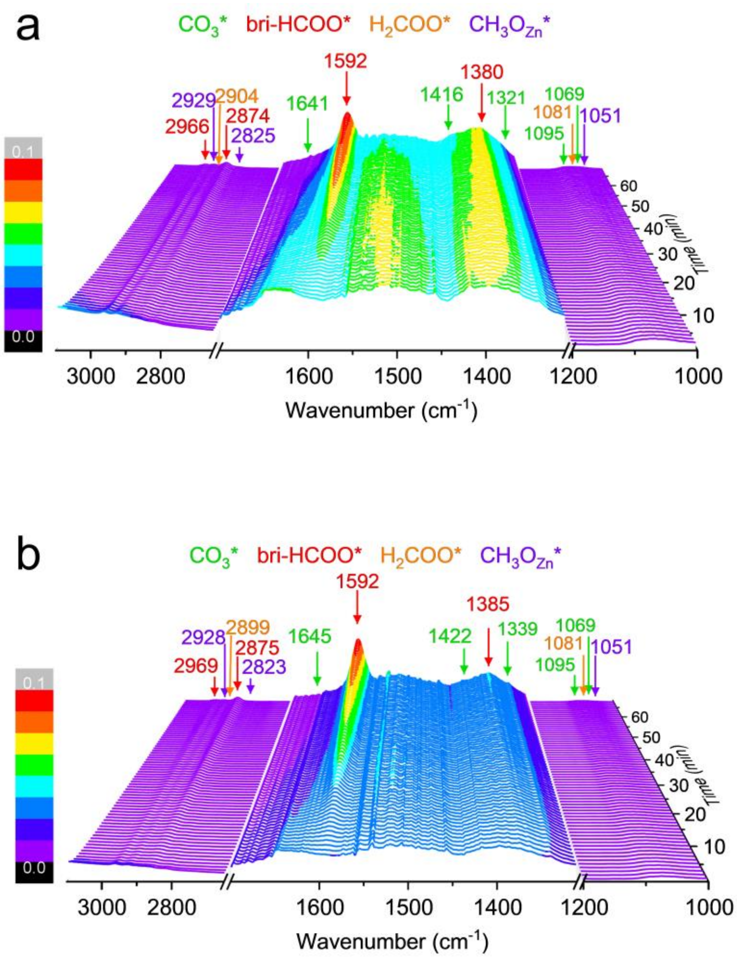

Supplementary Figure 29. temporal in situ DRIFTS spectra of Zn20Zr80 (pretreated in 3 MPa CO_2 at 303 K for 60 min, then purged in Ar) exposed to 3 MPa H_2 at **a**, 443 K and **b**, 453 K.

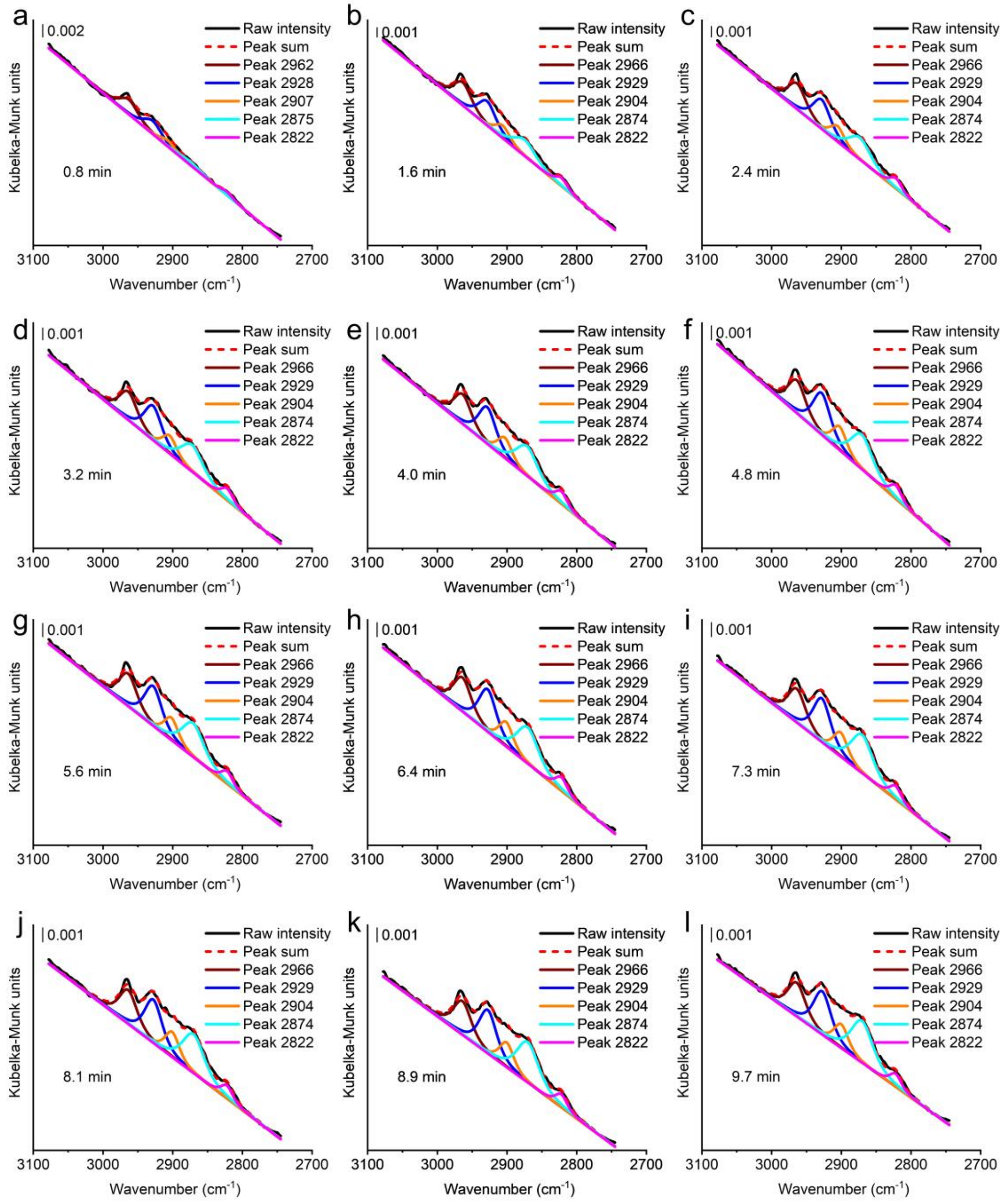

Supplementary Figure 30. a to **l**, peak-fitted time-resolved in situ DRIFTS spectra of Zn20Zr80 exposed to 3 MPa H_2 (pretreated in 3 MPa CO_2 at 303 K for 60 min, then purged in Ar) at 443 K.

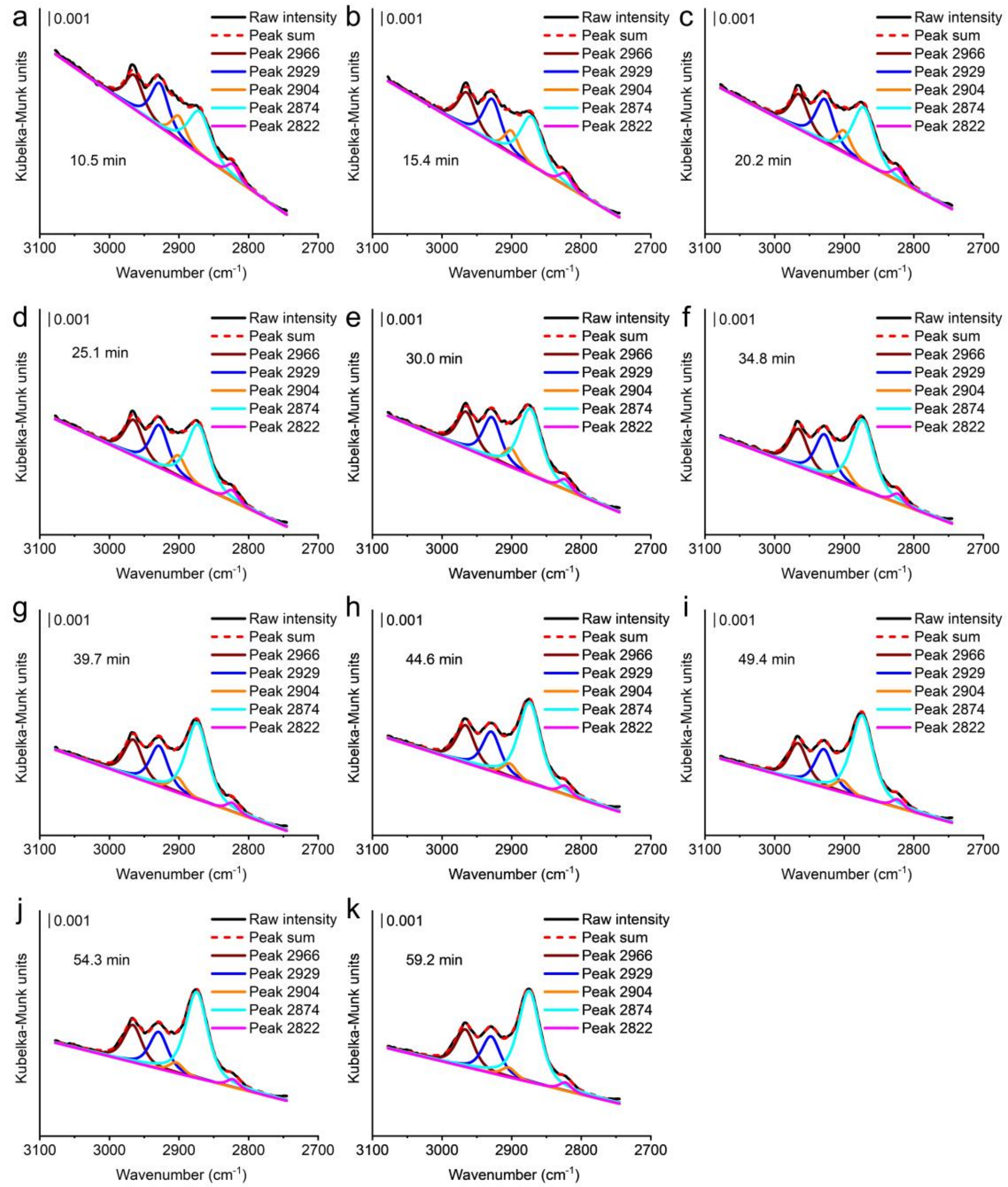

Supplementary Figure 31. a to **k**, peak-fitted time-resolved in situ DRIFTS spectra of Zn20Zr80 exposed to 3 MPa H_2 (pretreated in 3 MPa CO_2 at 303 K for 60 min, then purged in Ar) at 443 K.

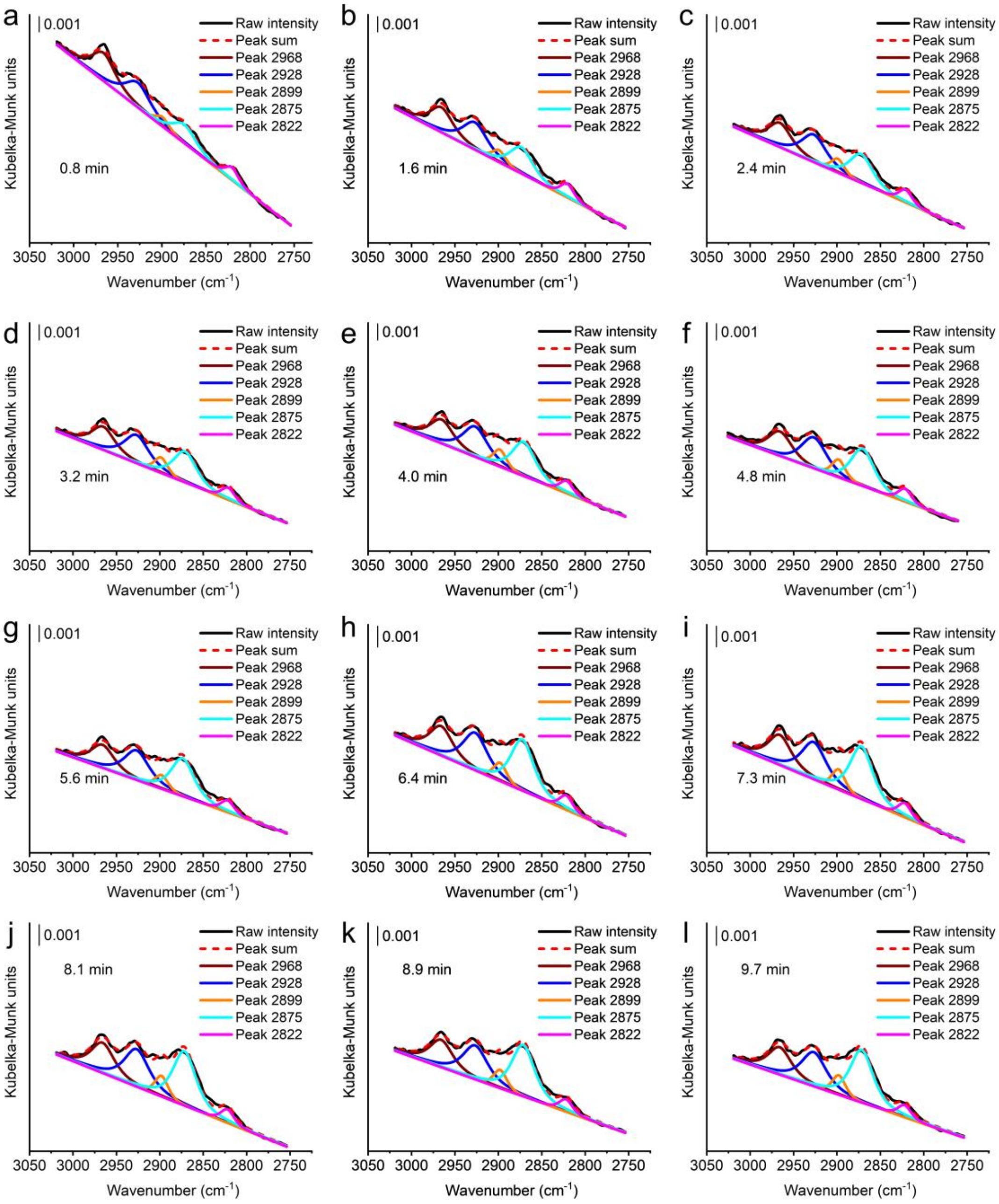

Supplementary Figure 32. a to **l**, peak-fitted time-resolved in situ DRIFTS spectra of Zn20Zr80 exposed to 3 MPa H_2 (pretreated in 3 MPa CO_2 at 303 K for 60 min, then purged in Ar) at 453 K.

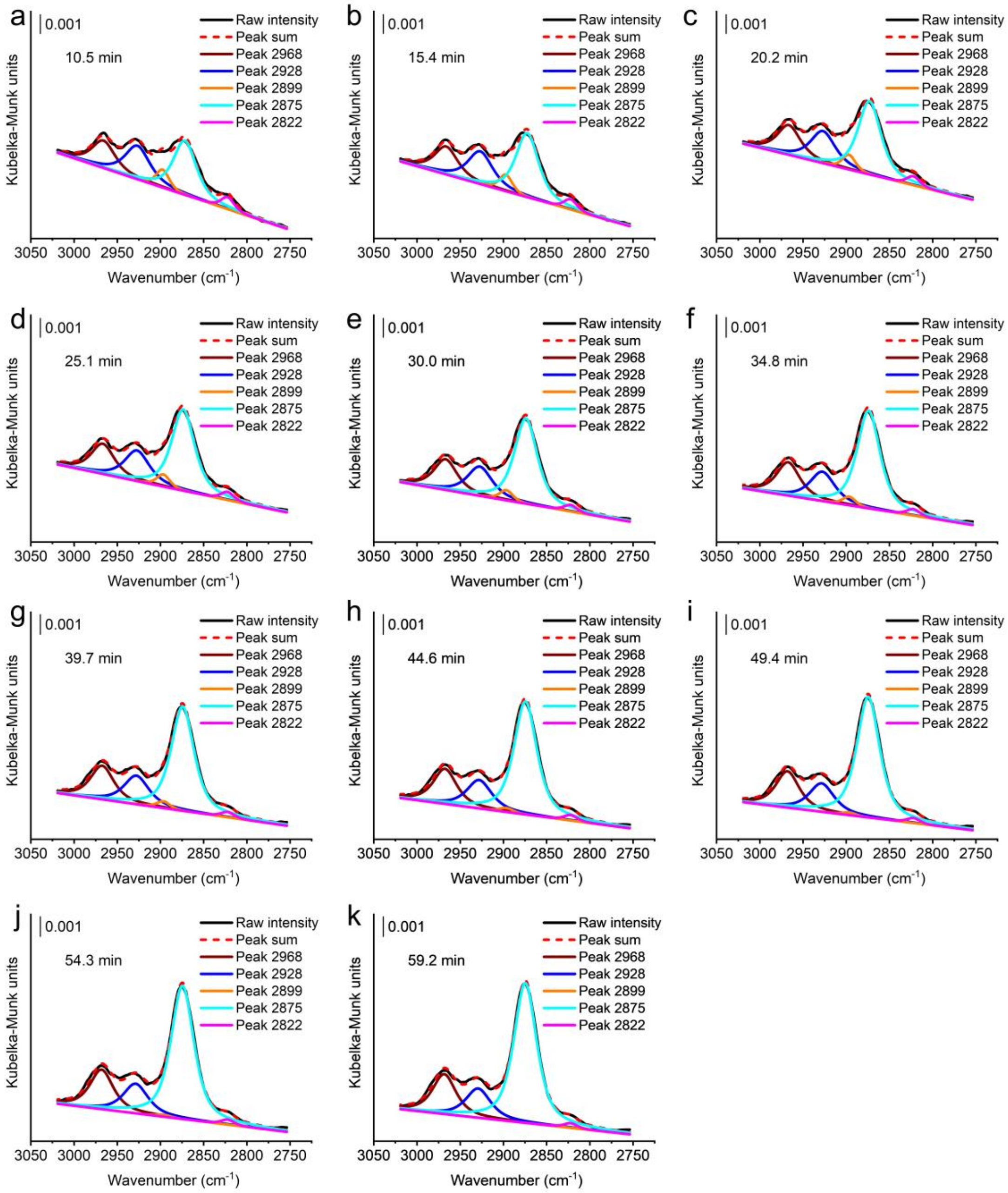

Supplementary Figure 33. a to **k**, peak-fitted time-resolved in situ DRIFTS spectra of Zn20Zr80 exposed to 3 MPa H_2 (pretreated in 3 MPa CO_2 at 303 K for 60 min, then purged in Ar) at 453 K.

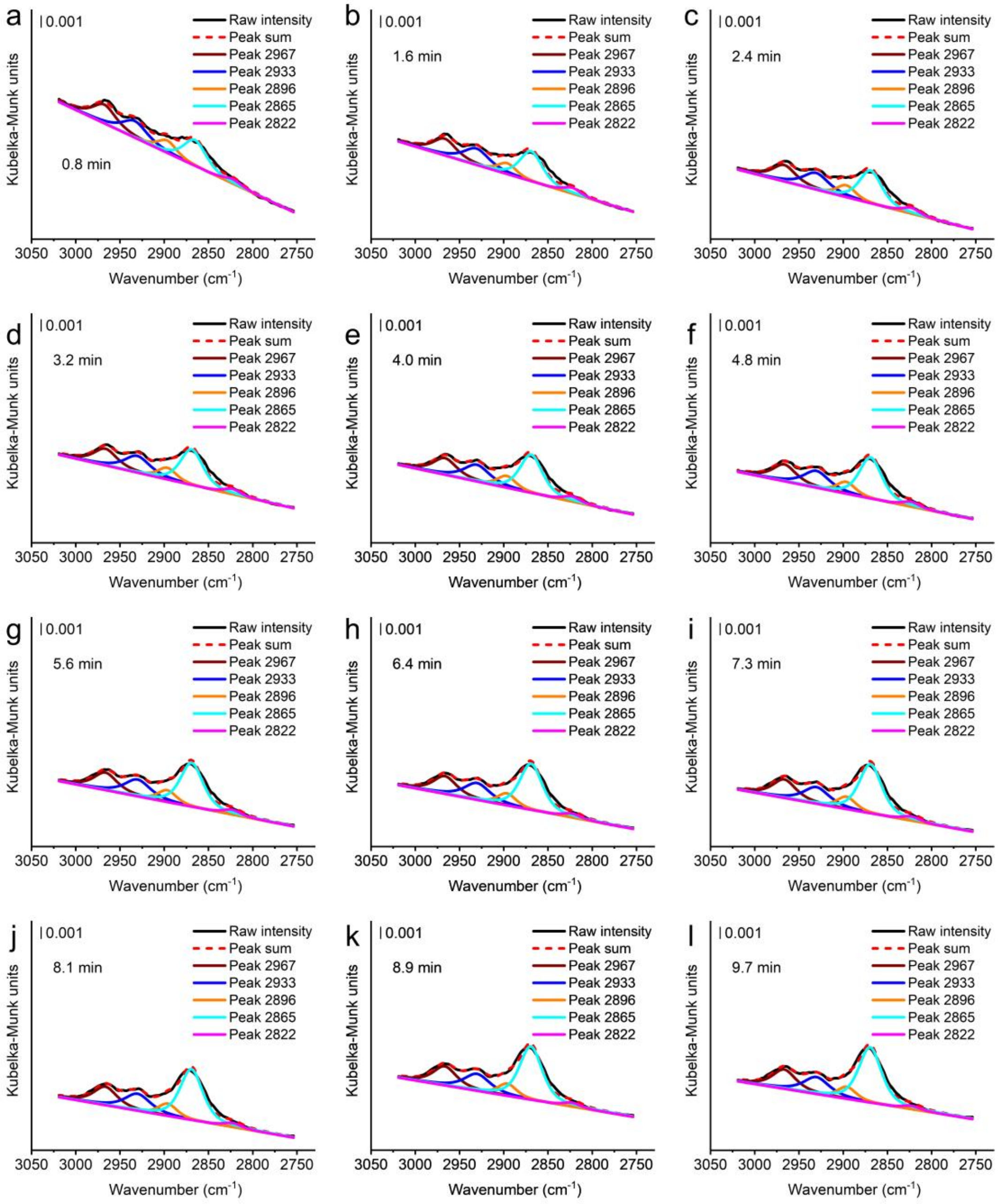

Supplementary Figure 34. a to **l**, peak-fitted time-resolved in situ DRIFTS spectra of Zn20Zr80 exposed to 3 MPa H_2 (pretreated in 3 MPa CO_2 at 303 K for 60 min, then purged in Ar) at 463 K.

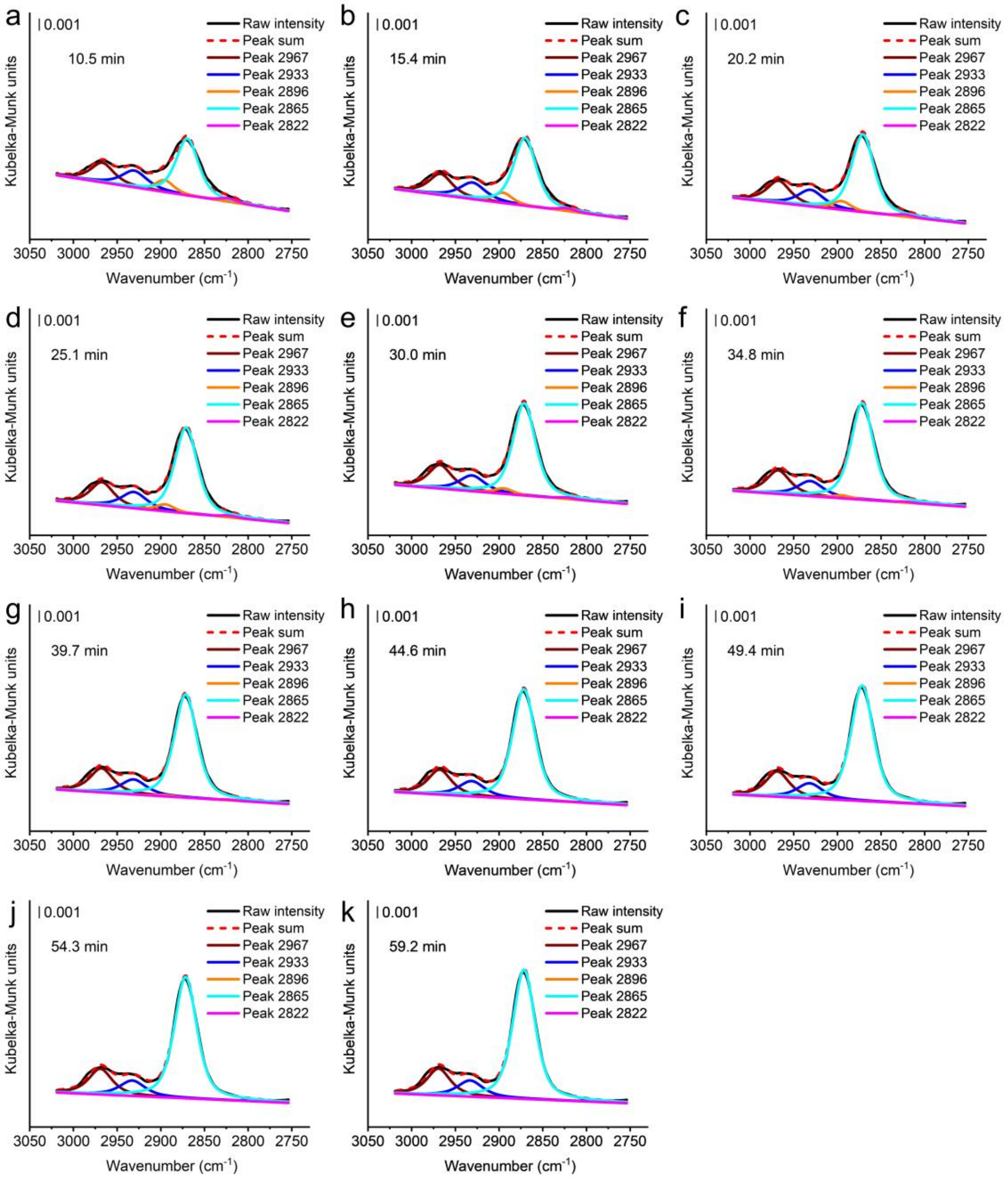

Supplementary Figure 35. a to **k**, peak-fitted time-resolved in situ DRIFTS spectra of Zn20Zr80 exposed to 3 MPa H_2 (pretreated in 3 MPa CO_2 at 303 K for 60 min, then purged in Ar) at 463 K.

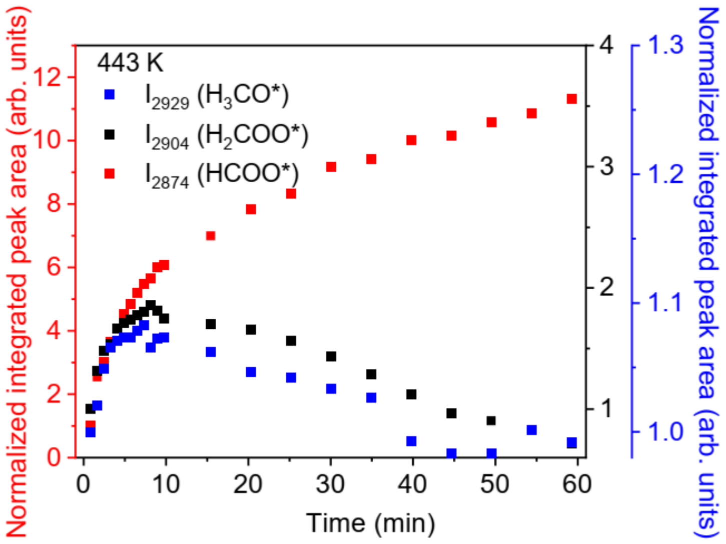

Supplementary Figure 36. The corresponding normalized integrated peak area of various species in Supplementary Figure 29a as a function of time on Zn20Zr80 catalyst at 443 K.

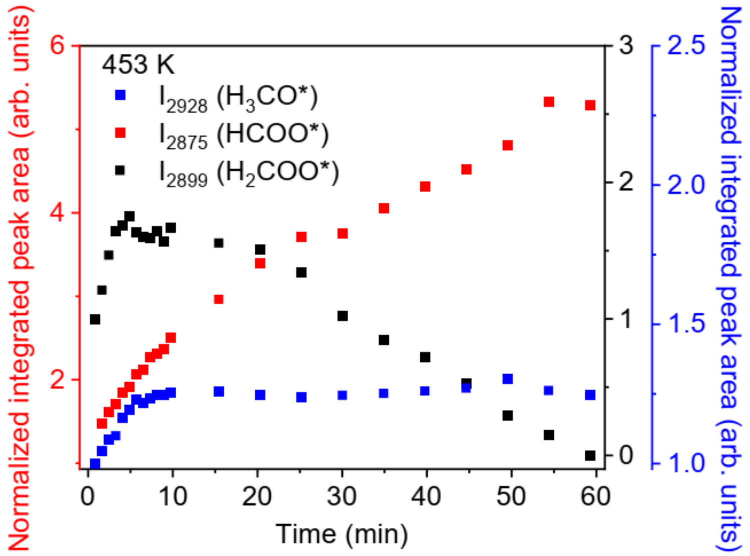

Supplementary Figure 37. The corresponding normalized integrated peak area of various species in Supplementary Figure 29b as a function of time on Zn20Zr80 catalyst at 453 K.

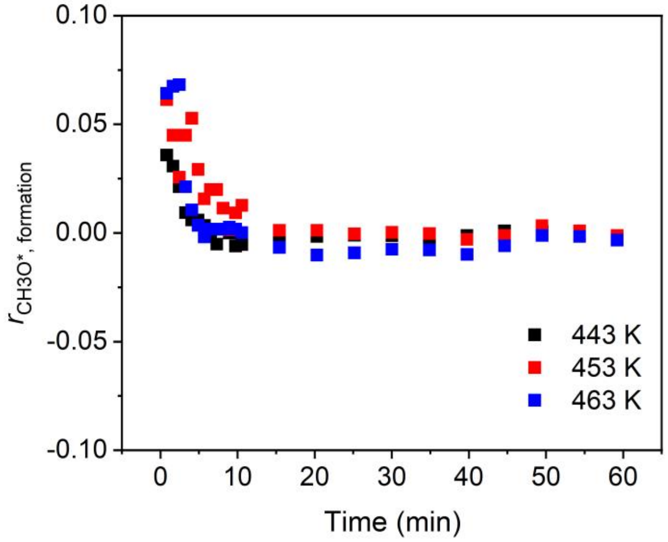

Supplementary Figure 38. The rate of CH_3O^* formation reaction on Zn20Zr80 at 443 K, 453 K and 463 K as a function of time derived from Fig. 4e, as well as Supplementary Figure 36 to 37.

$CO_2 * + H * \xrightarrow{r_1} HCOO *$ in equilibrium

$HCOO \xrightarrow{r_{-1}} CO_2 * + H *$

$HCOO * + H * \xrightarrow{r_2} H_2COO *$ in equilibrium

$H_2COO * \xrightarrow{r_{-2}} HCOO * + H *$

$H_2COO * + 2H * \xrightarrow{r_3} CH_3O * + OH *$ in equilibrium

$CH_3O * + OH * \xrightarrow{r_{-3}} H_2COO * + 2H *$

$CH_3O * + H * \xrightarrow{r_4} CH_3OH *$ rate-limiting step

$CH_3OH * \xrightarrow{r_5} CH_3OH(g)$ in equilibrium

$CH_3OH(g) \xrightarrow{r_{-5}} CH_3OH *$

Supplementary Figure 39. Elementary surface reaction kinetic of low-temperature surface reaction pathway with the CH_3O* hydrogenation as the rate-limiting elementary surface reaction.

$$r_{CH_3O*,app} = -\frac{d[CH_3O *]}{dt} = r_3 + r_{-3} + r_4$$

From our experimental data (Fig. 4f and Supplementary Figure 36 to 37) that the CH_3O* coverage keeps increasing within 5 min, suggesting that during the initial reaction time, the CH_3O* coverages are low so that its reactions to generate CH_3OH* or H_2COO* barely occur, i.e., both r_{-3} and r_4 are much smaller than r_3.

Then, during the initial CH_3O* formation reaction,

$$r_{CH_3O*,app} = -\frac{d[CH_3O *]}{dt} = r_3$$

Assuming that the relative surface coverage of CH_3O* is proportional to the area of its IR vibrational peak at 2925 cm^{-1}, then,

$$-\frac{d[I_{2925\ cm^{-1}}]}{dt} = r_{3,app}$$

Thus, the $r_{3,app}$ can be derived from the data of $[I_{2925\ cm^{-1}}]_t$ versus t during the initial CH_3O* formation reaction (Supplementary Figure 38), and the activation energy of CH_3O* formation reaction can be calculated using $r_{3,app}$ data at different reaction temperatures.

Supplementary Table 4. Listed data includes the compositions, structure, DFT energy, NN energy and energy differences between DFT energy and NN energy (E_{diff}, meV/atom)

No.	Species	N_{atom}	E_{DFT}/eV	E_{nn}/eV	$\Delta(E_{nn}-E_{DFT})$/(meV/atom)
1	$Zr_{59}O_{118}$(slab)	177	-1656.687	-1656.628	0.326
2	$Zn_1Zr_{59}O_{119}$(GM)	179	-1665.768	-1664.707	5.922
3	$Zn_1Zr_{59}O_{119}$ (2nd lowest minimum)	179	-1665.546	-1664.479	5.587
4	$Zn_2Zr_{58}O_{118}$(GM)	178	-1646.367	-1645.315	5.890
5	$Zn_3Zr_{57}O_{117}$(GM)	177	-1626.516	-1625.473	5.876
	RMS				4.720

Supplementary Table 5. Relative potential energies (ΔE), free energies (ΔG) and local snapshots of initial state (IS), transition state (TS) and final state (FS) of each elementary step of CO_2 hydrogenation reactions on Zn_1-ZrO_2 catalyst. The energy of clean surface is set to zero. All the energies are in the unit of kJ mol^{-1}.

	**Zn_1-ZrO_2**		
	**IS**	**TS**	**FS**
	H^*+CO_2* → bri-$HCOO^*$		
	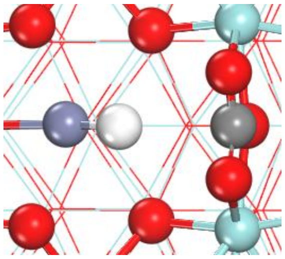	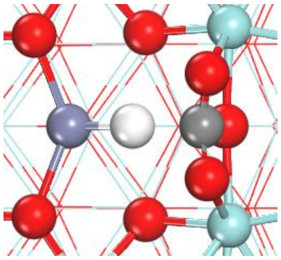	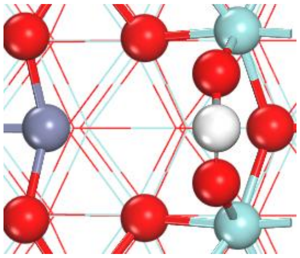
ΔE	-115.8	-77.2	-256.7
ΔG	44.4	79.1	-98.4
	H^*+ bri-$HCOO^*$ → H_2COO^*		
	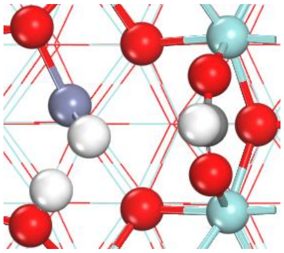	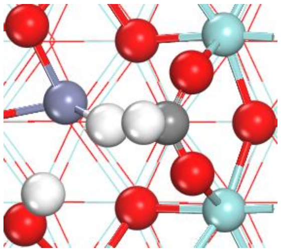	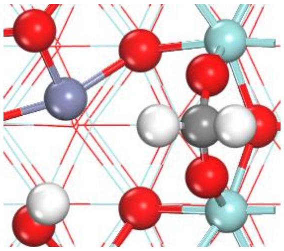
ΔE	-286.6	-250.9	-276.9
ΔG	-73.3	-42.5	-62.7
	H_2^*+ H_2COO^*→ H_2COOH^*		
	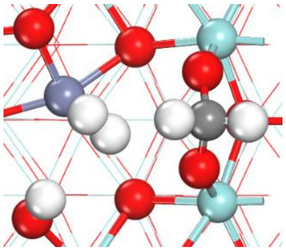	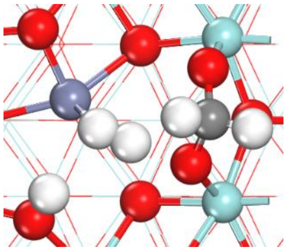	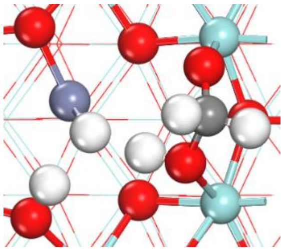
ΔE	-281.7	-270.2	-303.0
ΔG	-14.5	-2.9	-35.7
	H^*+ H_2COOH^*→ H_3CO^*+OH^*		

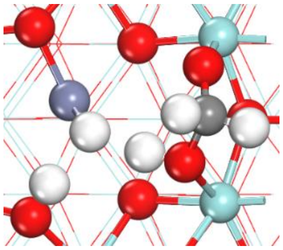 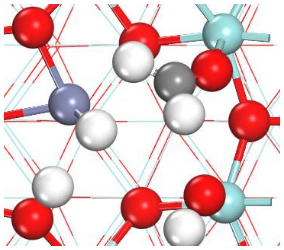 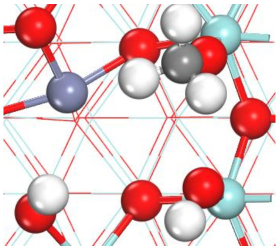

ΔE	-303.0	-225.8	-345.4
ΔG	-35.7	-40.5	-77.2

$OH^{*}+H^{*}\rightarrow H_2O^{*}$

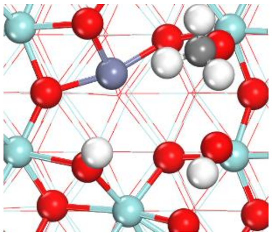 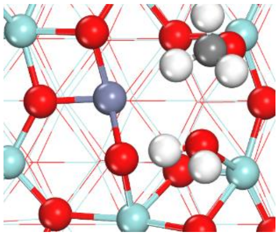 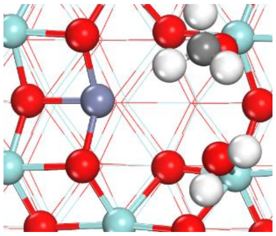

ΔE	-345.4	-311.6	-330.0
ΔG	-77.2	-45.3	-61.8

$H^{*}+H_3CO^{*} \rightarrow H_3COH^{*}$

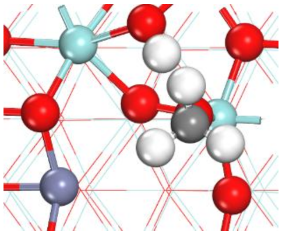 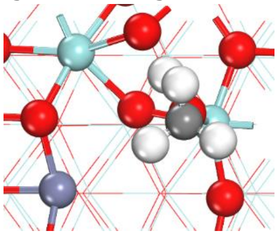 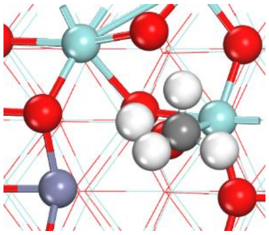

ΔE	-330.0	-238.3	-250.9
ΔG	-61.8	25.1	17.4

Supplementary Table 6. Relative potential energies (ΔE), free energies (ΔG) and local snapshots of initial state (IS), transition state (TS) and final state (FS) of each elementary step of CO_2 hydrogenation reactions on Zn_3-ZrO_2 catalyst. The energy of clean surface is set to zero. All the energies are in the unit of kJ mol^{-1}.

	**Zn_3-ZrO_2**		
	**IS**	**TS**	**FS**
	H^*+CO_2* → bri-$HCOO^*$		
	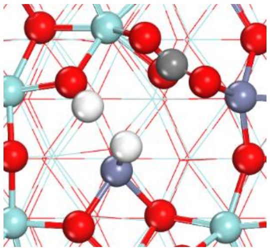	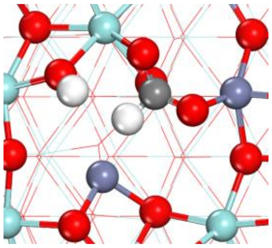	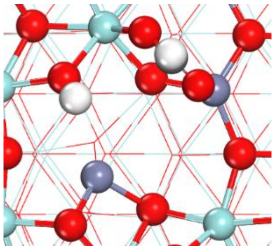
ΔE	-125.4	-36.7	-158.2
ΔG	-33.8	122.5	-1.9
	H^*+ bri-$HCOO^*$ → H_2COO^*		
	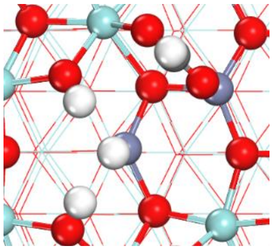	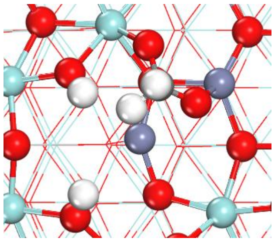	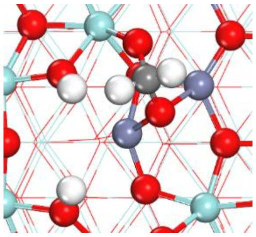
ΔE	-234.5	-84.9	-155.3
ΔG	-20.3	126.4	57.9
	H^*+ H_2COO^* → H_2COOH^*		
	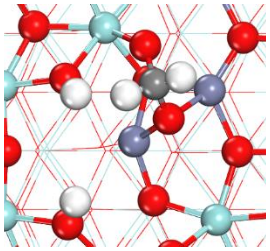	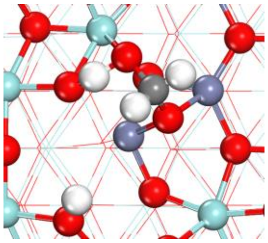	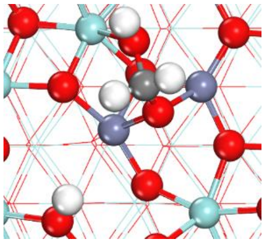
ΔE	-155.3	-111.0	-153.4
ΔG	57.9	96.5	59.8
	H^*+ H_2COOH^* → H_3CO^*+OH^*		

	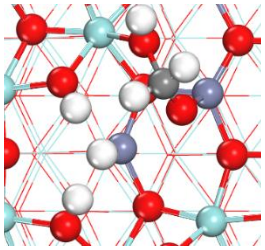	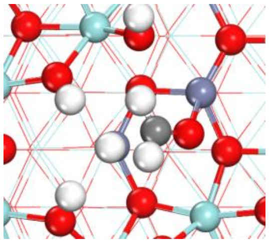	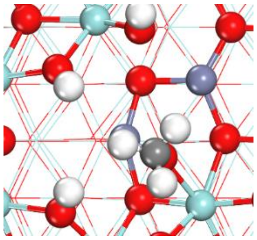
ΔE	-186.2	-170.8	-278.8
ΔG	81.0	95.5	-11.6
		$H^* + OH^* \rightarrow H_2O^*$	
	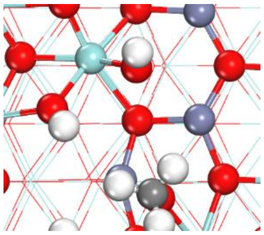	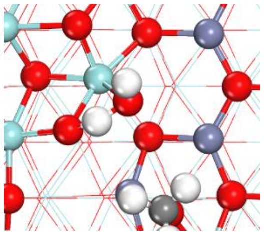	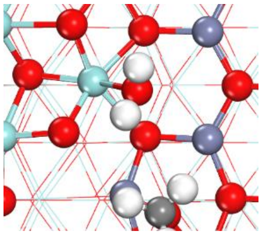
ΔE	-278.8	-232.5	-245.1
ΔG	-11.6	32.8	22.2
		$H^* + H_3CO^* \rightarrow H_3COH^*$	
	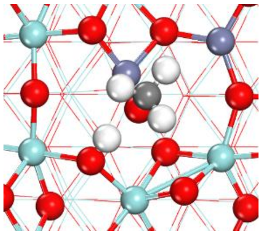	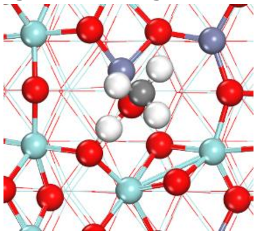	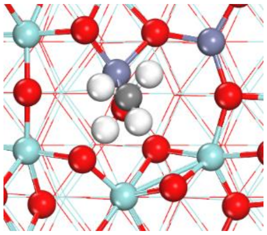
ΔE	-245.1	-242.2	-248.9
ΔG	22.2	25.1	18.3

Supplementary Table 7. The computed transition state energy (G_a, kJ mol^{-1}) of CO_2 hydrogenation to methanol reaction using PBE functional with without and with D3 dispersion correction (PBE-D3).

	w/o. d3	w. d3
G_a (TS1)	34.73	30.88
G_a (TS2)	30.88	28.95
G_a (TS3)	10.61	8.68
G_a (TS4)	76.22	80.08
G_a (TS5)	31.84	27.98
G_a (TS6)	86.84	82.98
G_a (TS1’)	88.77	86.84
G_a (TS2’)	150.52	151.48
G_a (TS3’)	44.38	46.31
G_a (TS4’)	16.40	13.51
G_a (TS5’)	46.31	48.24
G_a (TS6’)	2.89	2.89

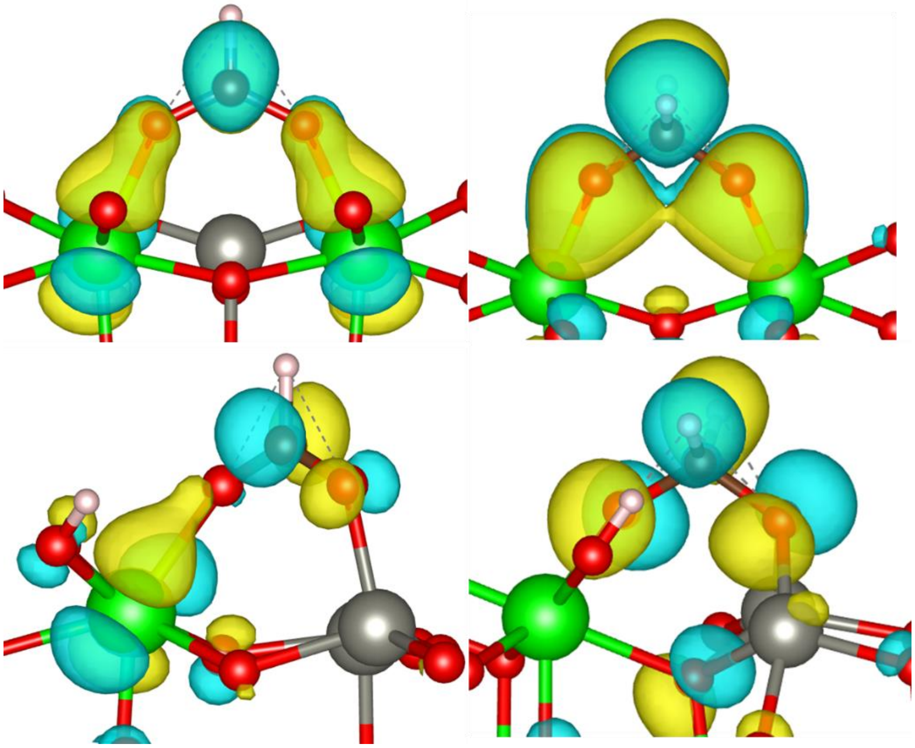

Supplementary Figure 40. The Wavefunction of HCOO and H_2COO on $GM_{8.3}$(1Zn) and GM_{25}(3Zn). The color scheme for atoms is Zr: green ball; Zn: gray ball; O: red ball; H: white ball; C: brown ball.

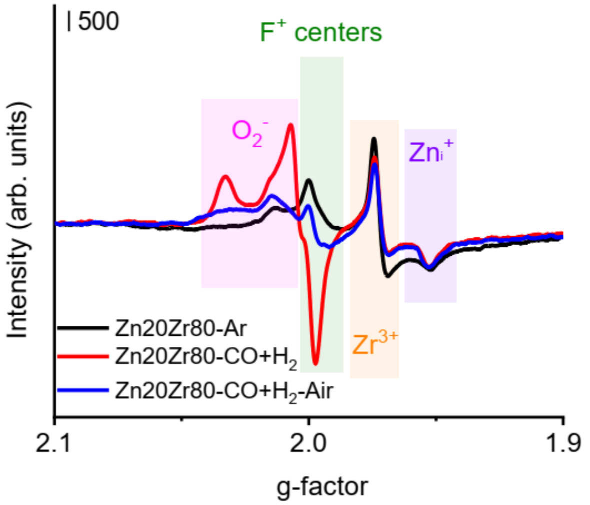

Supplementary Figure 41. EPR spectra of Zn20Zr80 calcined in Ar at 773 K without exposure to air (black curve), and Zn20Zr80 calcined in Ar at 773 K and subsequently treated under 3 MPa $CO+H_2$ at 573 K without exposure to air (red curve), and then exposed to the air for 10 min (blue curve). The signals appearing at 2.030/2.014/2.005, 2.000, 1.947 and 1.957 arise from O_2^- [28-30], F^+ centers[30-34], Zr^{3+} [29,34,35] and Zn_i^+ [36,37], respectively.

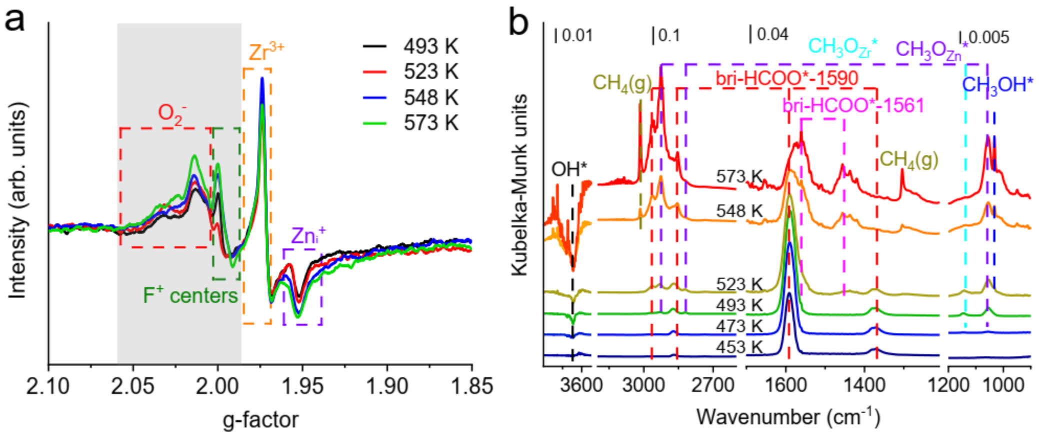

Supplementary Figure 42. a, steady-state in situ DRIFTS spectra of Zn20Zr80 under 3 Mpa CO+H_2 at indicated temperatures. **b,** EPR spectra of Zn20Zr80 calcined in Ar at 773 K and subsequently treated under 3 MPa CO+H_2 at indicated temperatures without exposure to air.

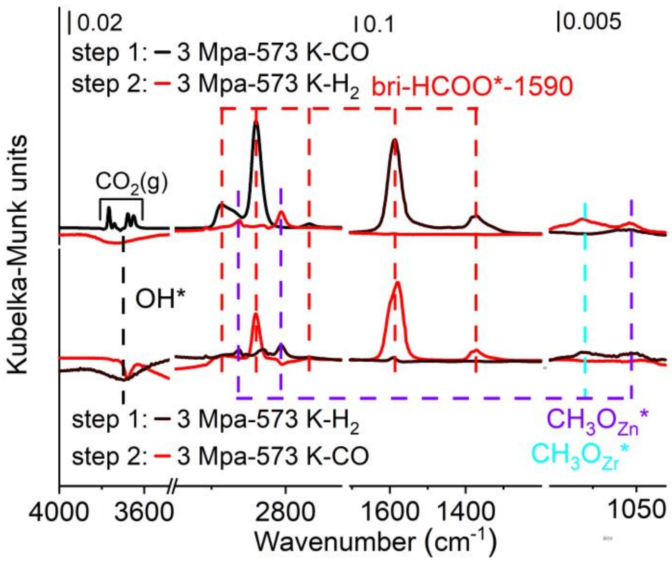

Supplementary Figure 43. steady-state DRIFTS spectra of Zn20Zr80 first exposed to 3 MPa CO at 573 K, then purged in Ar and exposed to 3 MPa H_2 at 573 K and first exposed to 3 MPa H_2, then purged in Ar and exposed to 3 MPa CO under 573 K.

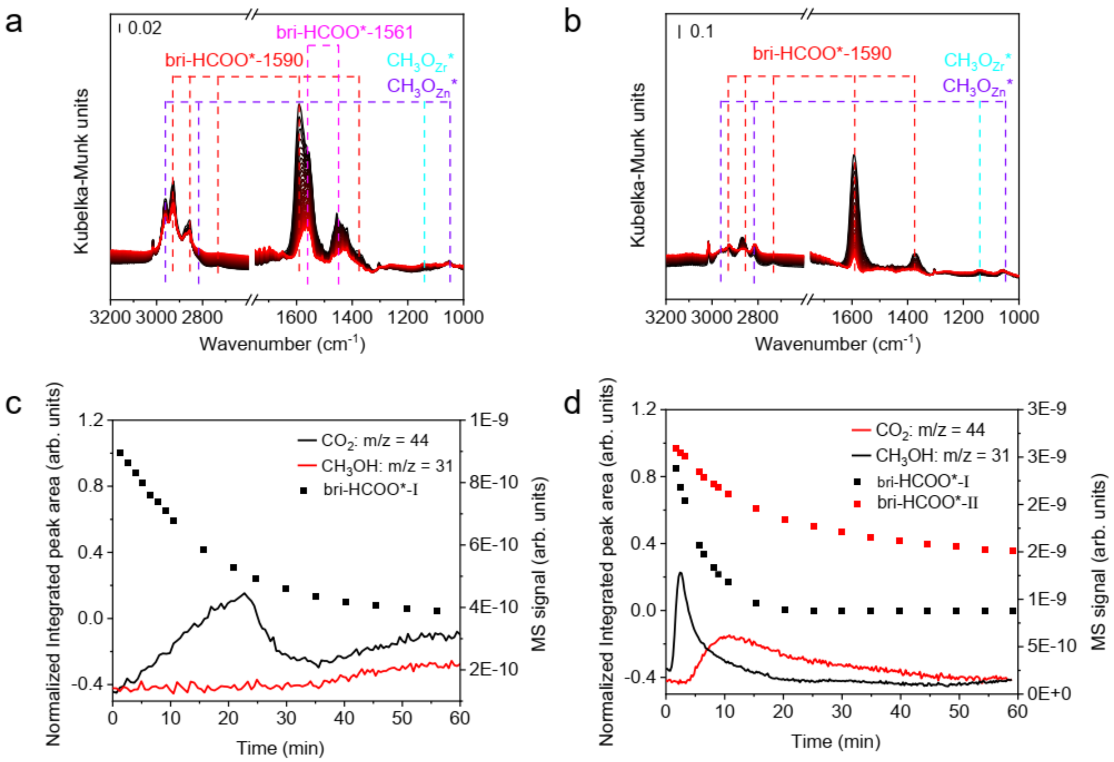

Supplementary Figure 44. a, temporal in situ DRIFTS spectra of Zn20Zr80 exposed to 3 MPa H_2 (pretreated in 3 MPa CO+H_2 (H_2: CO = 2) at 453 K for 60 min, then purged in Ar) at 573 K. **b**, temporal in situ DRIFTS spectra of Zn20Zr80 exposed to 3 MPa H_2 (pretreated in 3 MPa CO+H_2 (H_2: CO_2 = 2) at 573 K for 60 min, then purged in Ar) at 573 K. **c**, The normalized integrated peak area of formate species and MS signal of CH_3OH and CO_2 as a function of time on Zn20Zr80 catalyst exposed to 3 MPa H_2 (pretreated under 3 Mpa CO+H_2 at 453 K and purged with Ar) at 573 K. **d**, The normalized integrated peak area of bri-HCOO*-1590 and bri-HCOO*-1561 species and MS signal of CH_3OH and CO_2 as a function of time on Zn20Zr80 catalyst exposed to 3 MPa H_2 (pretreated under 3 Mpa CO+H_2 at 573 K and purged with Ar) at 573 K.

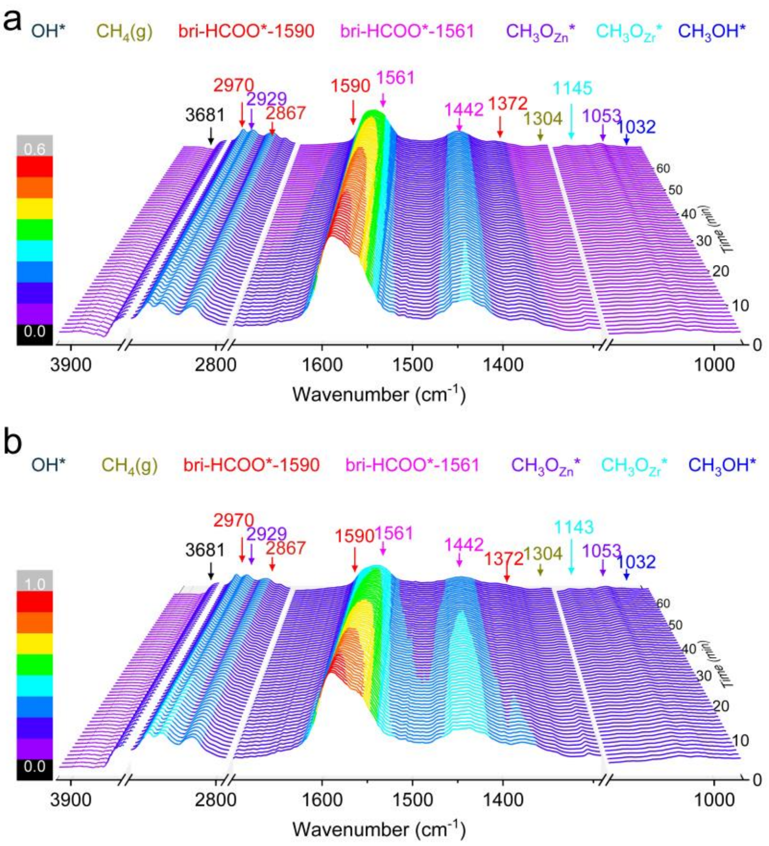

Supplementary Figure 45. temporal in situ DRIFTS spectra of Zn20Zr80 (pretreated in 3 MPa CO+H_2 (H_2: CO = 2) at 573 K for 60 min, then purged in Ar) exposed to 3 MPa H_2 at **a**, 523 K and **b**, 548 K.

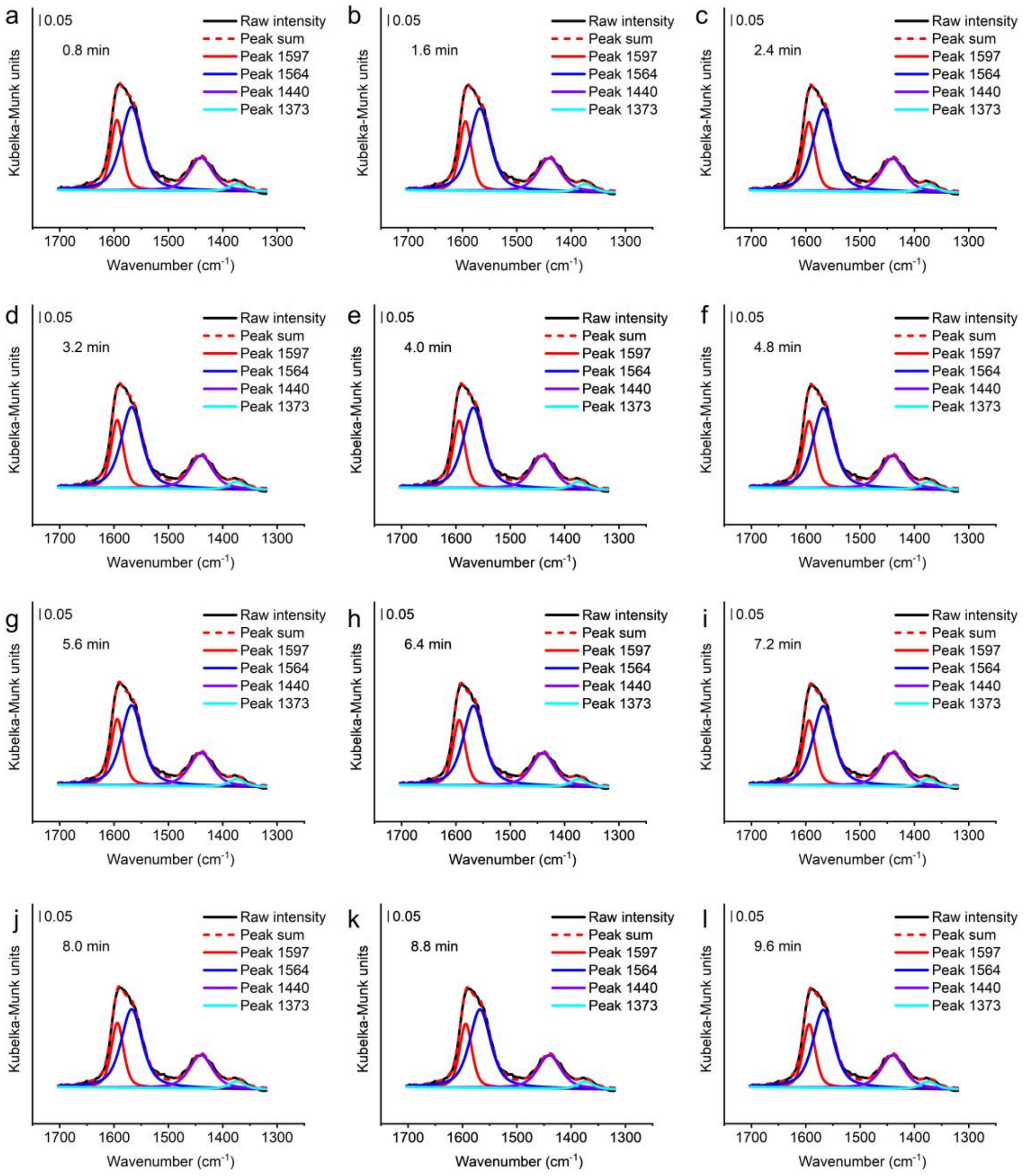

Supplementary Figure 46. a, to **l**, peak-fitted temporal in situ DRIFTS spectra of Zn20Zr80 (pretreated in 3 MPa CO+H_2 (H_2: CO = 2) at 573 K for 60 min, then purged in Ar) exposed to 3 MPa H_2 at 523 K.

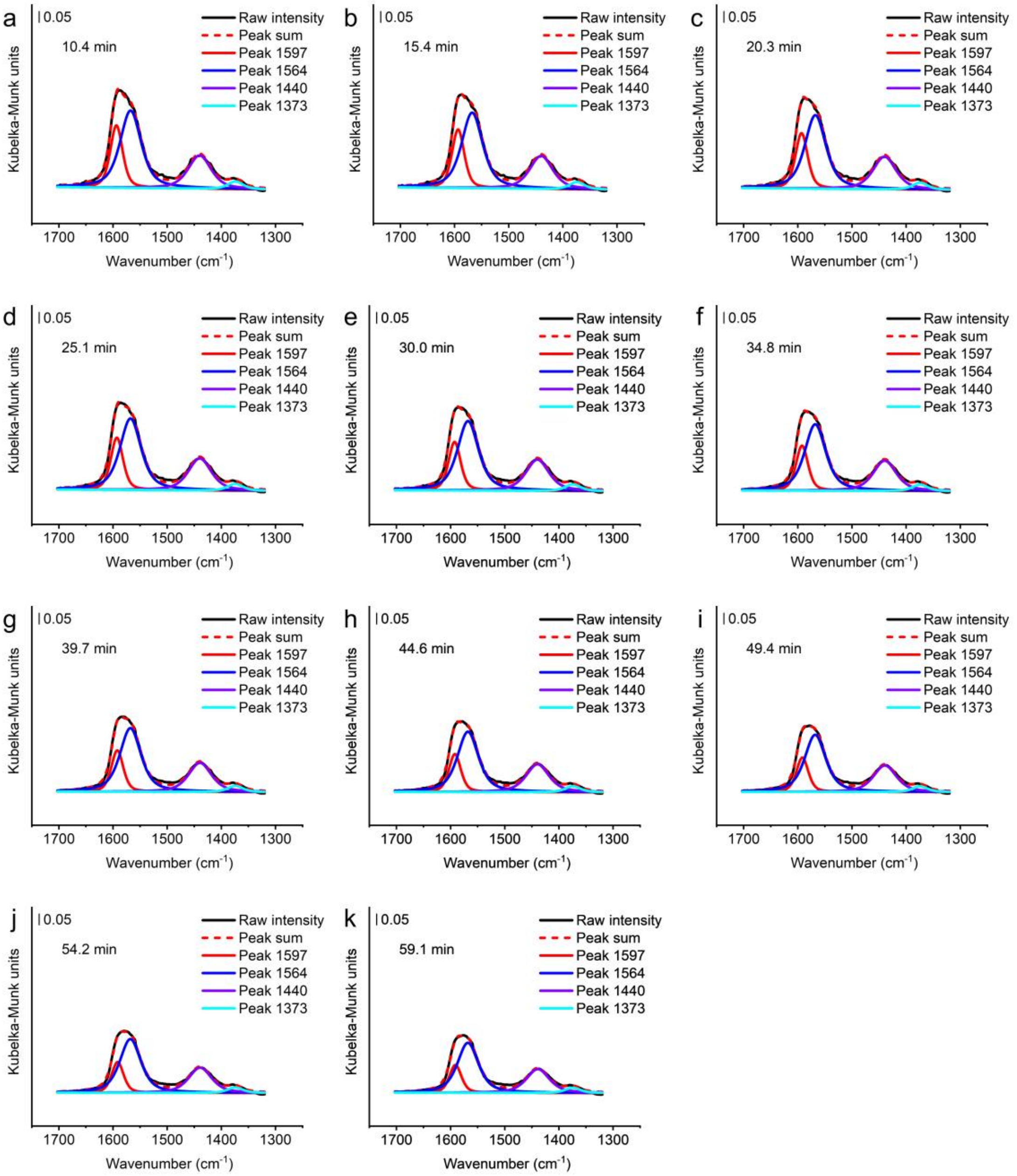

Supplementary Figure 47. a, to **k**, peak-fitted temporal in situ DRIFTS spectra of Zn20Zr80 (pretreated in 3 MPa CO+H_2 (H_2: CO = 2) at 573 K for 60 min, then purged in Ar) exposed to 3 MPa H_2 at 523 K.

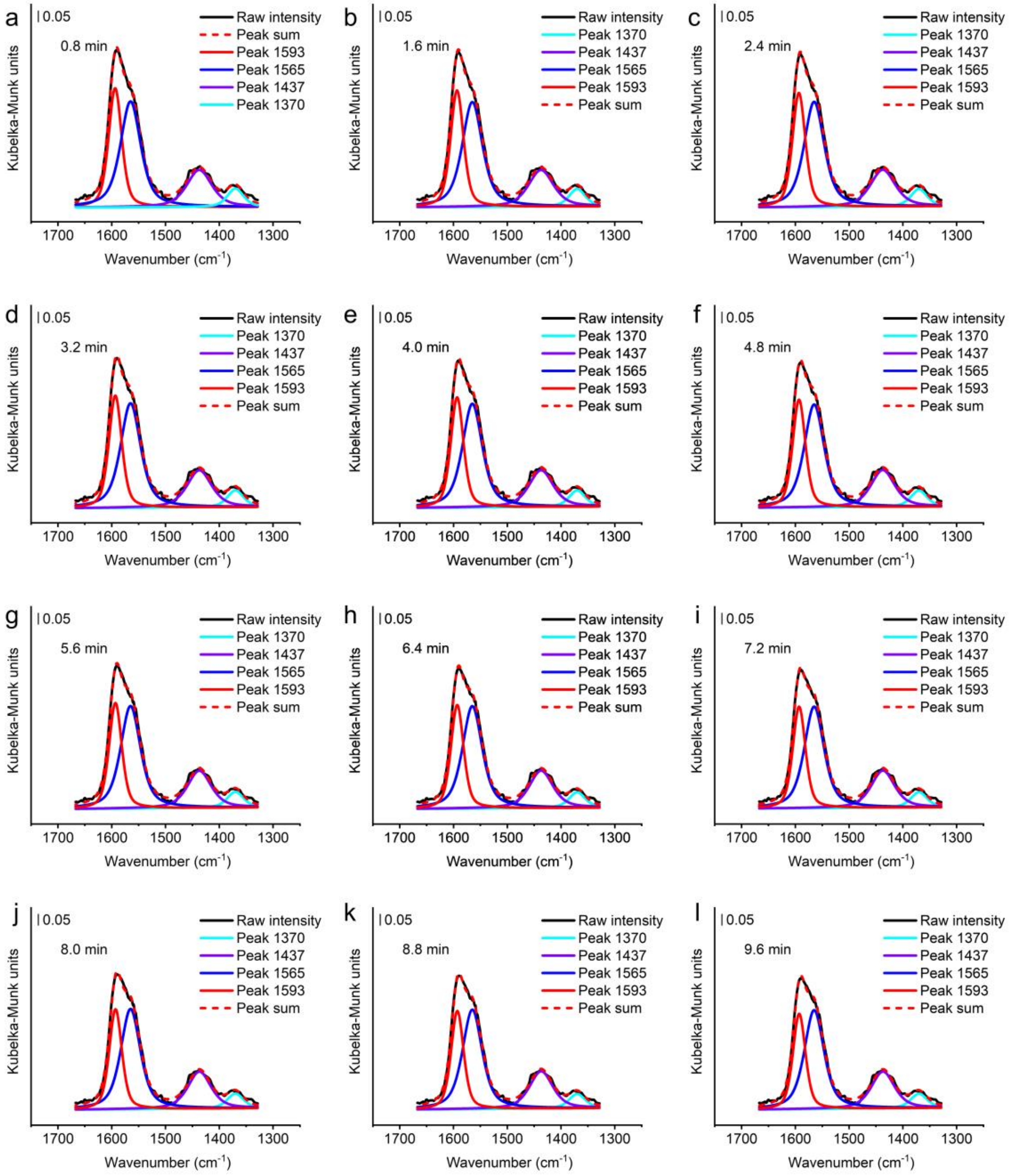

Supplementary Figure 48. a, to **l**, peak-fitted temporal in situ DRIFTS spectra of Zn20Zr80 (pretreated in 3 MPa CO+H_2 (H_2: CO = 2) at 573 K for 60 min, then purged in Ar) exposed to 3 MPa H_2 at 548 K.

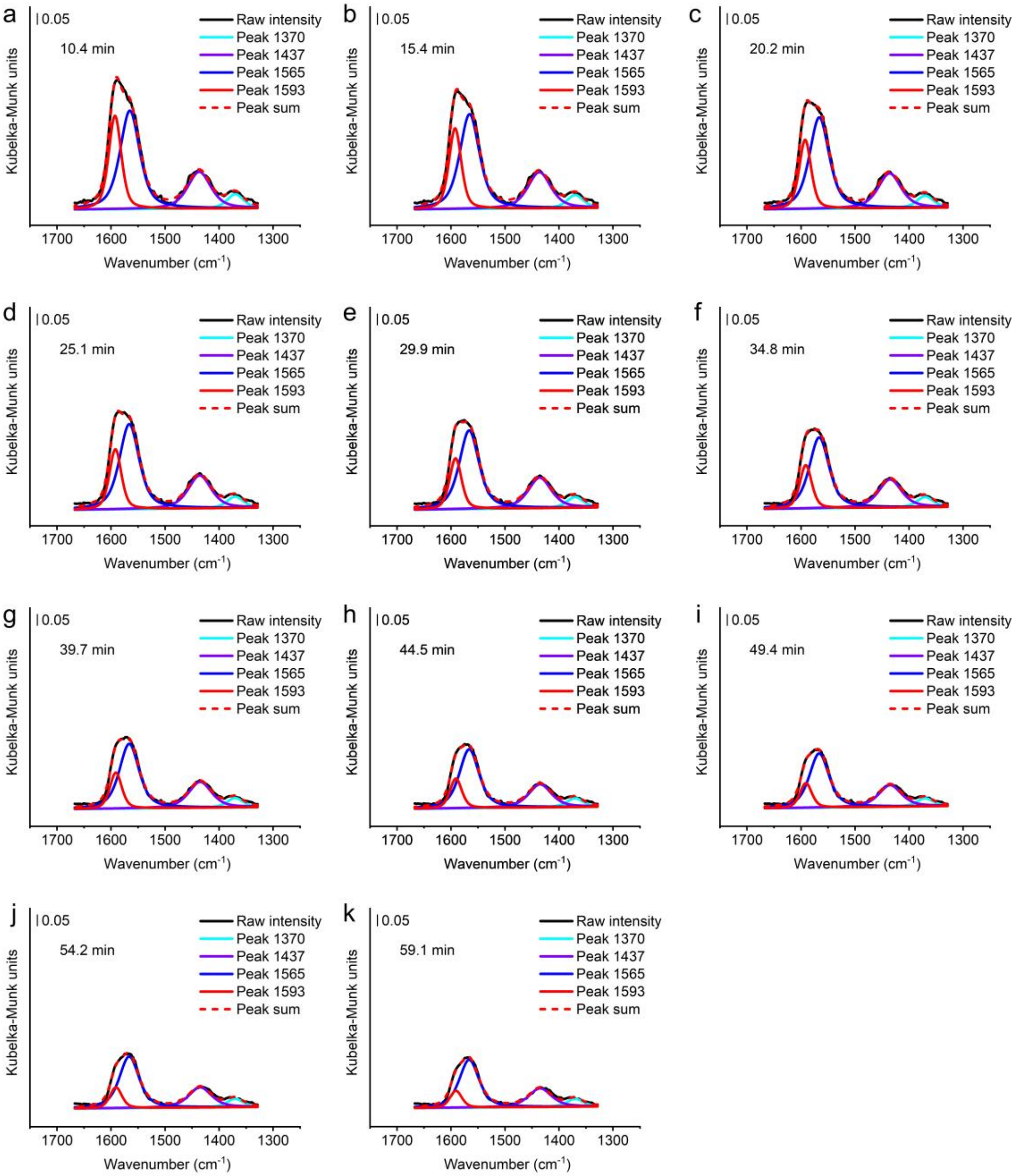

Supplementary Figure 49. a, to **k**, peak-fitted temporal in situ DRIFTS spectra of Zn20Zr80 (pretreated in 3 MPa CO+H_2 (H_2: CO = 2) at 573 K for 60 min, then purged in Ar) exposed to 3 MPa H_2 at 548 K.

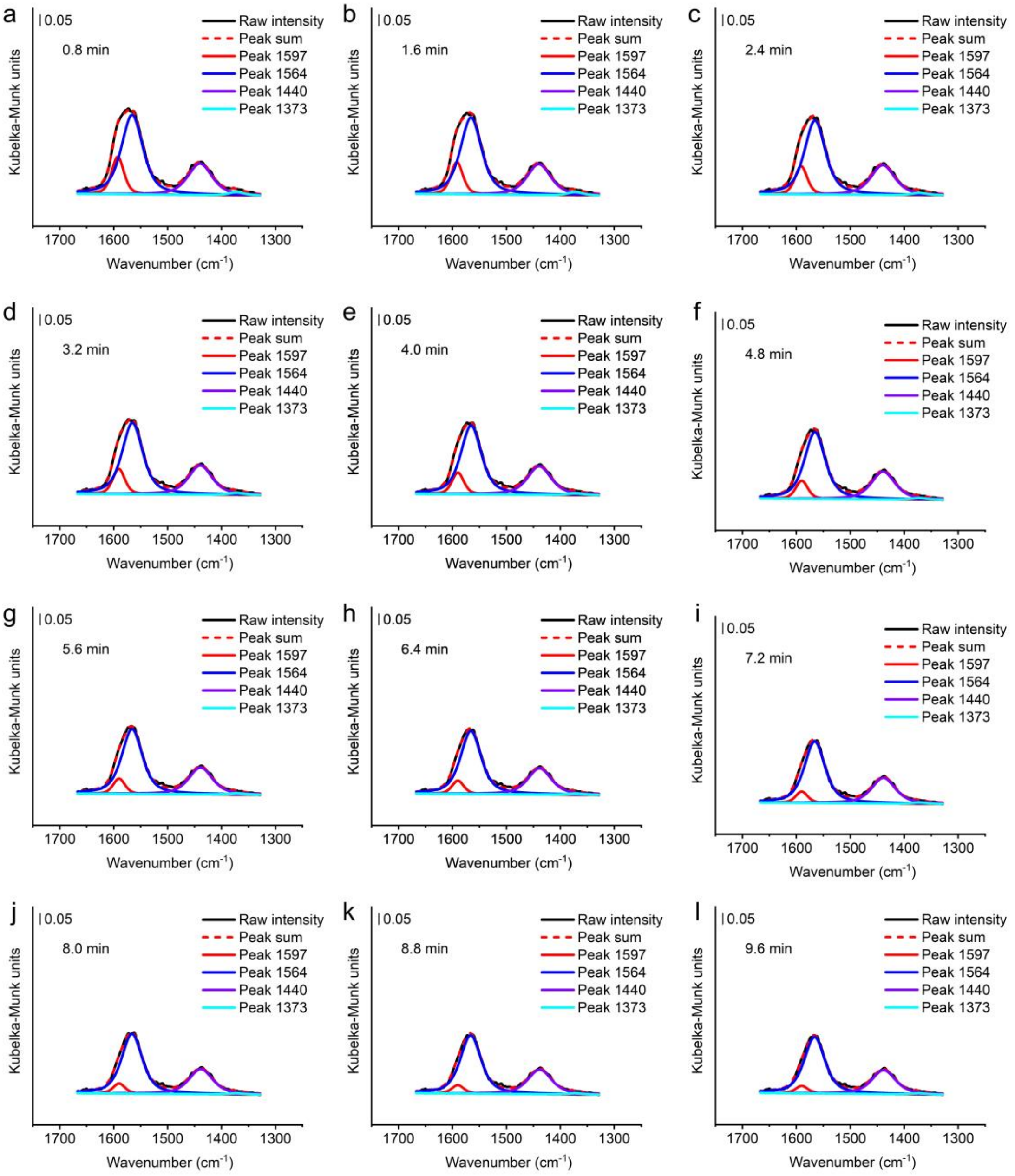

Supplementary Figure 50. a, to **l**, peak-fitted temporal in situ DRIFTS spectra of Zn20Zr80 (pretreated in 3 MPa CO+H_2 (H_2: CO = 2) at 573 K for 60 min, then purged in Ar) exposed to 3 MPa H_2 at 573 K.

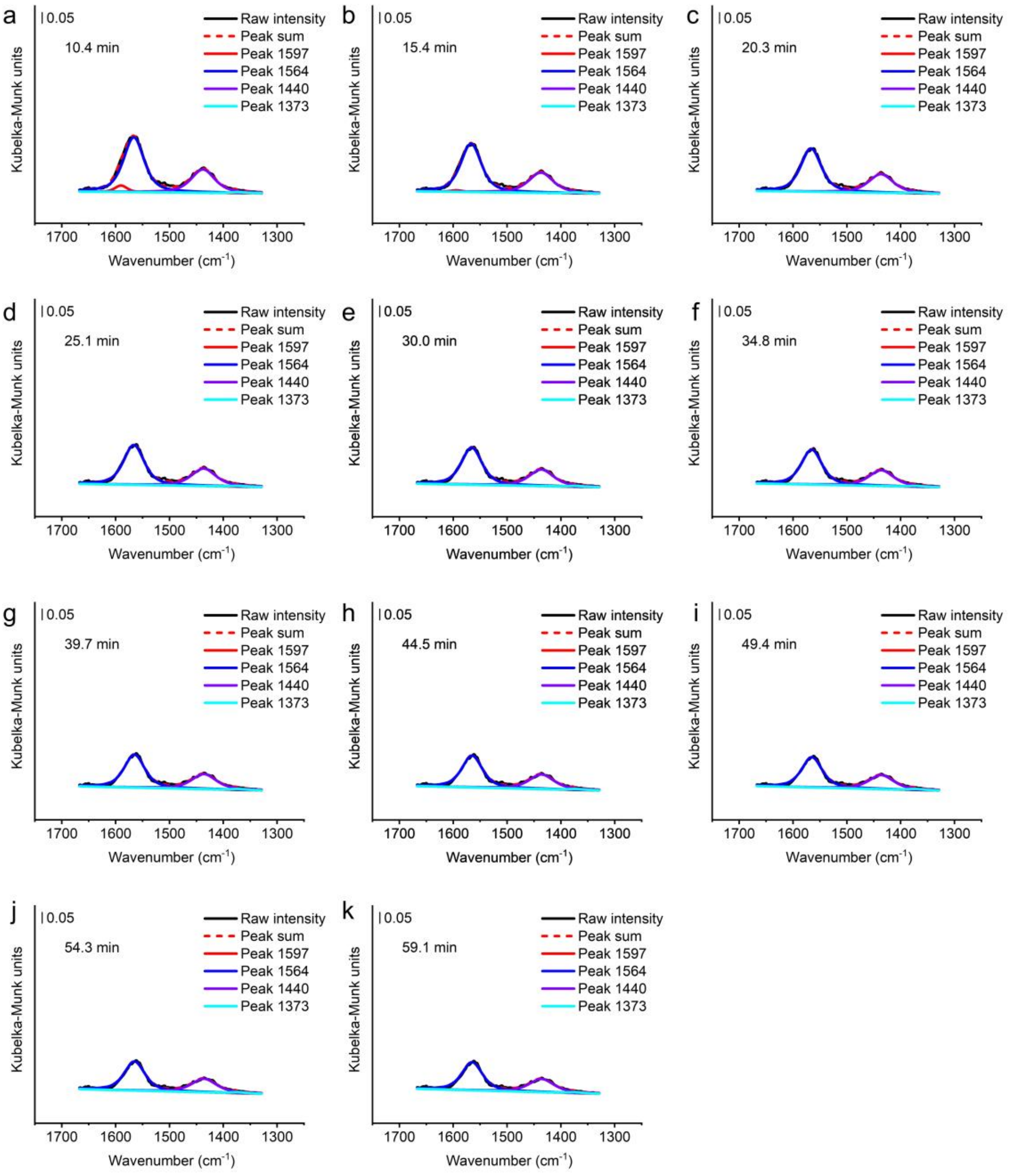

Supplementary Figure 51. a, to **k**, peak-fitted temporal in situ DRIFTS spectra of Zn20Zr80 (pretreated in 3 MPa CO+H_2 (H_2: CO = 2) at 573 K for 60 min, then purged in Ar) exposed to 3 MPa H_2 at 573 K.

$$HCOO^* + 2H^* \xrightarrow{r_1} CH_3O^*$$ rate-limiting step

$$CH_3O^* + H^* \xrightarrow{r_2} CH_3OH^*$$ in equilibrium

$$CH_3OH^* \xrightarrow{r_{-2}} CH_3O^* + H^*$$

$$CH_3OH^* \xrightarrow{r_3} CH_3OH(g)$$ in equilibrium

$$CH_3OH(g) \xrightarrow{r_{-3}} CH_3OH^*$$

Supplementary Figure 52. Elementary surface reaction kinetic starting from HCOO* with the HCOO* hydrogenation reaction as the rate-limiting step.

$$r_{HCOO^*} = -\frac{d[HCOO^*]}{dt} = r_1 = k_1 \times [H^*]^2 \times [HCOO^*]$$

During the hydrogenation of HCOO* in 3 MPa H_2, the [H*] can be considered as a constant.

Thus,

$$r_{HCOO*} = \; -\frac{d[HCOO\,*]}{dt} = k_{1,app} \times [HCOO\,*]$$

Assuming that the relative surface coverage of HCOO* is proportional to the area of its IR vibrational peaks at 1560 and 1590 cm^{-1},

$$r_{HCOO^*,app} = -\frac{d[I_{1590}]}{dt} = r_{1,app,1590} = k_{1,app,1590} \times [I_{1590}]$$

$$r_{HCOO^*,app} = -\frac{d[I_{1560}]}{dt} = r_{1,app,1560} = k_{1,app,1560} \times [I_{1560}]$$

then, $ln\frac{[I_{1590}]_t}{[I_{1590}]_0} = -k_{1,app,1590} \times t,\; ln\frac{[I_{1560}]_t}{[I_{1560}]_0} = -k_{1,app,1560} \times t$

Thus, $k_{1,app,1590}$ and $k_{1,app,1560}$ are the slopes of the fitted lines of ln $\frac{[I_{1590}]_t}{[I_{1590}]_0}$ versus *t* and of ln $\frac{[I_{1560}]_t}{[I_{1560}]_0}$) versus *t* as shown in Figure 7c and Figure 7d. Also, the $r_{1,app,1590}$ and $r_{1,app,1560}$ can be derived from the data of$[I_{1590}]_t$ and $[I_{1560}]_t$versus *t* during the HCOO* hydrogenation

reaction (Supplementary Figure 54). Then, the activation energy of HCOO* hydrogenation reaction can be calculated using either $k_{1,app,1590}$ and $k_{1,app,1560}$ or $r_{1,app,1590}$ and $r_{1,app,1560}$ data at different reaction temperatures (Fig. 7e and Supplementary Figure 56).

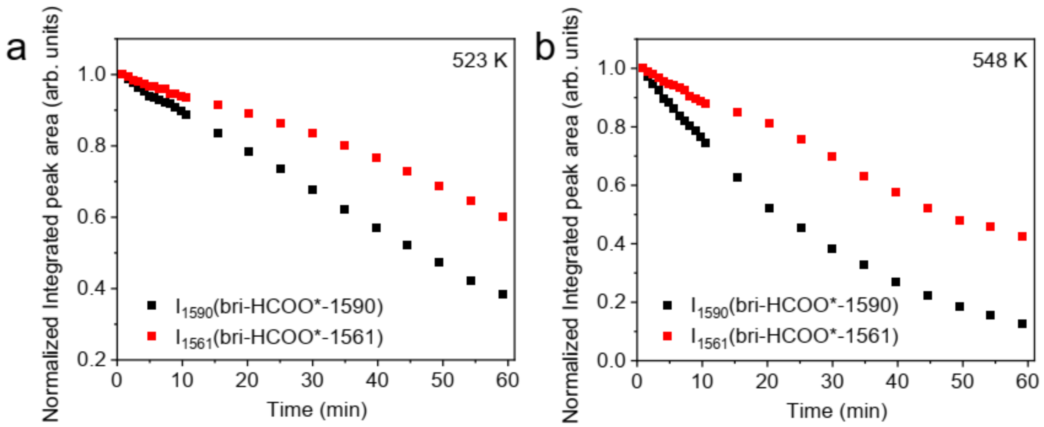

Supplementary Figure 53. The corresponding normalized integrated peak area of observed species in Supplementary Figure 45 and Figure 7a as a function of time on Zn20Zr80 catalyst at **a**, 523 K, **b**, 548 K.

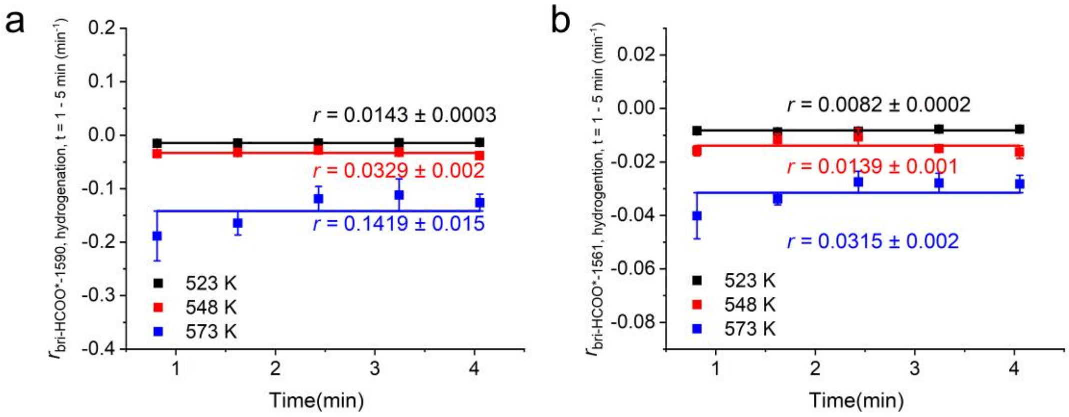

Supplementary Figure 54. a, the average rate of bri-HCOO*-1590 and **b,** bri-HCOO*-1561 hydrogenation reaction on Zn20Zr80 at 523 K, 548 K and 573 K at 1 to 5 min derived from Supplementary Figure 53 and Fig. 7a. The error bars in the figure represent the standard errors (SE) of the fitted values.

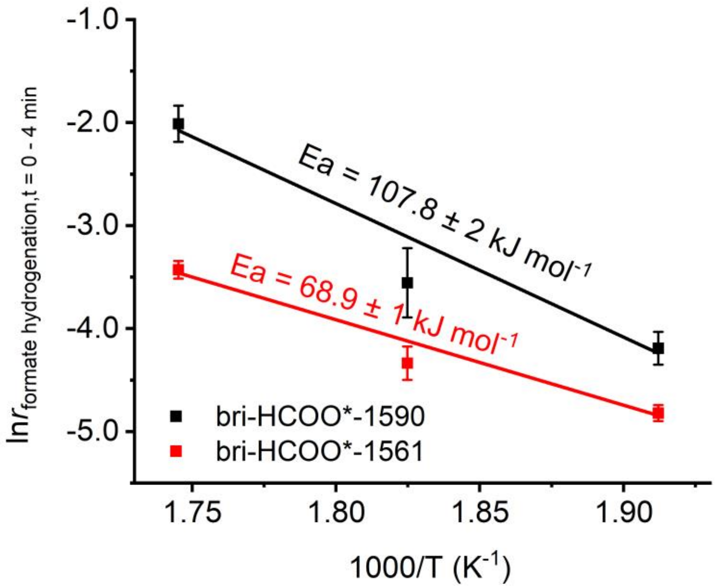

Supplementary Figure 55. Arrhenius plots of bri-HCOO*-1590 and bri-HCOO*-1561 hydrogenation reaction on Zn20Zr80 derived from Supplementary Figure 54. The error bars in the figure represent the standard errors (SE) of the fitted values.

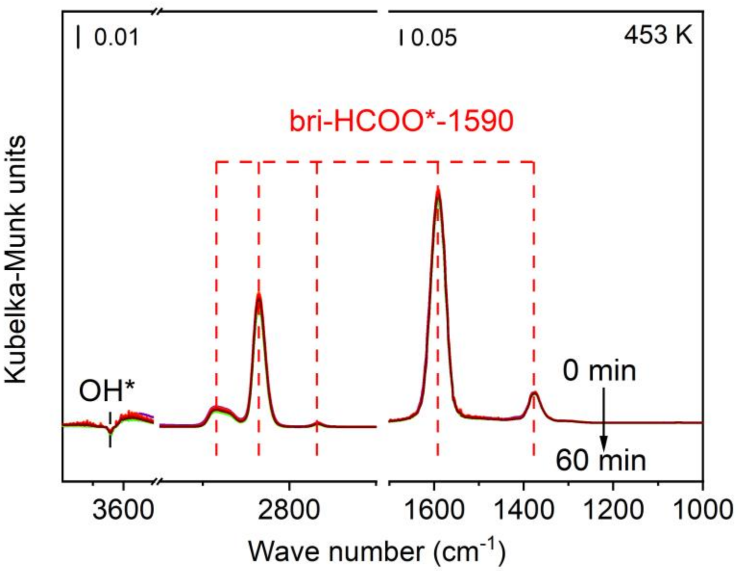

Supplementary Figure 56. Temporal in situ DRIFTS spectra of Zn20Zr80 exposed to 3 MPa H_2 (pretreated in 3 MPa CO+H_2 (H_2: CO = 2) at 573 K for 60 min, then purged in Ar) at 453 K.

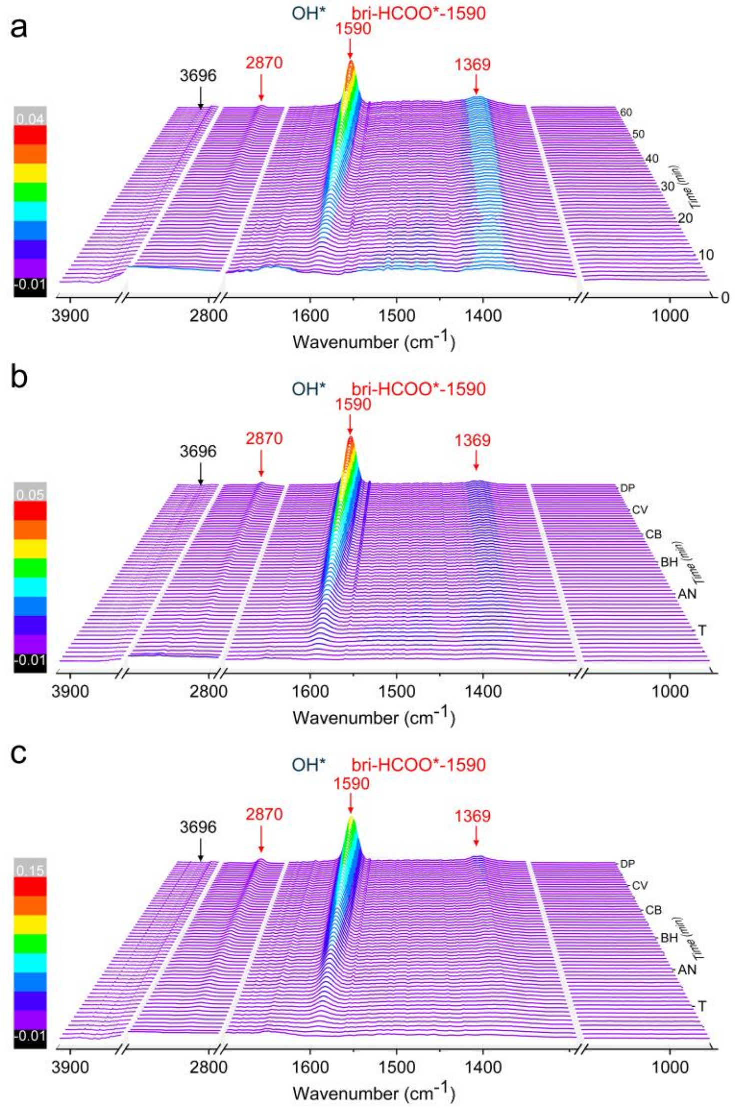

Supplementary Figure 57. Temporal in situ DRIFTS spectra of Zn20Zr80 exposed to 3 MPa CO+H_2 (H_2: CO=2:1) at (a) 423 K, (b) 433 K and (c) 443 K for 60 min.

$$CO^* + H^* + O_{br} \xrightarrow{r_1} HCOO^*$$

$$HCOO^* \xrightarrow{r_2} CO_2^* + H^*$$

$$HCOO^* + 2H^* \xrightarrow{r_3} CH_3O^* \quad \text{in equilibrium}$$

$$CH_3O^* \xrightarrow{r_{-3}} HCOO^* + 2H^*$$

Supplementary Figure 58. Elementary surface reaction kinetic starting from HCOO* formation reaction.

$$r_{HCOO^*,formation,app} = -\frac{d[HCOO^*]}{dt} = r_1 + r_2 + r_3 - r_{-3}$$

From our experimental data (Supplementary Figure 57) that the HCOO* coverage keeps unchanged within 60 min, suggesting that during the reaction time, the HCOO* coverages are stable so that its reactions to generate CH_3O^* or CO_2^* barely occur, i.e., Thus: $r_2 = r_3 = r_{-3} = 0$.

$$r_{HCOO^*,formation,app} = -\frac{d[HCOO^*]}{dt} = r_1$$

Assuming that the relative surface coverage of HCOO* and OH* is proportional to the area of its IR vibrational peaks at 1590 cm^{-1} and 3696 cm^{-1},

$$r_{HCOO^*,formation,app} = -\frac{d[I_{1590}]}{dt} = r_{1,app,1590} = k_{1,app,3696} \times [I_{3696}]$$

Then, $ln\frac{[I_{3696}]_t}{[I_{3696}]_0} = -k_{1,app,3696} \times t$

Thus, the $r_{1,app,1590}$ can be derived from the data of$[I_{1590}]_t$ versus *t* during the HCOO* formation reaction (Supplementary Figure 59 and 61). Also, $k_{1,app,3696}$ is the slopes of the fitted lines of ln $\frac{[I_{3696}]_t}{[I_{3696}]_0}$ versus *t* as shown in Supplementary Figure 62b. Then, the activation energy of bri-HCOO*-1590 formation reaction can be calculated using $r_{1,app,1590}$ and $k_{1,app,3696}$data at different reaction temperatures.

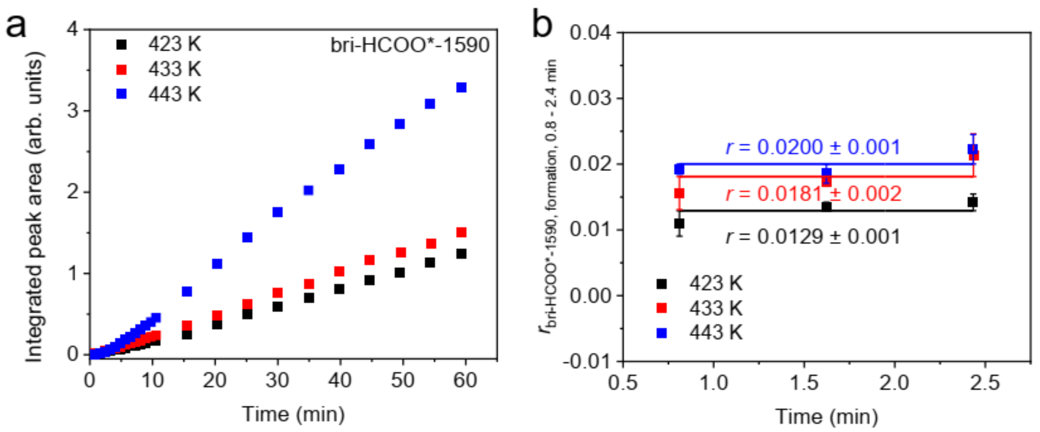

Supplementary Figure 59. a, the corresponding normalized integrated peak area of observed bri-HCOO*-1590 species in Supplementary Figure 57 as a function of time on Zn20Zr80 catalyst. **b,** the average rate of bri-HCOO*-1590 formation reaction on Zn20Zr80 at 0.8 to 2.5 min derived from Supplementary Figure 59a. The error bars in the figure represent the standard errors (SE) of the fitted values.

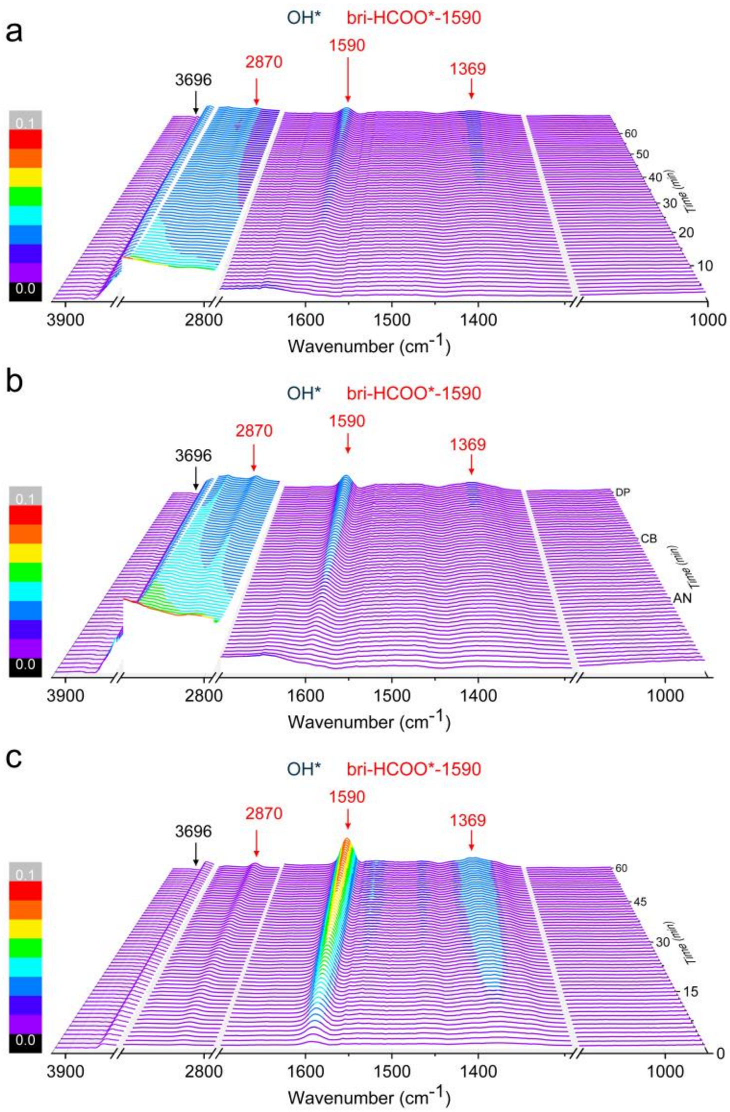

Supplementary Figure 60. Temporal in situ DRIFTS spectra of Zn20Zr80 exposed to 3 MPa CO at (a) 383 K, (b) 388 K and (c) 393 K for 60 min.

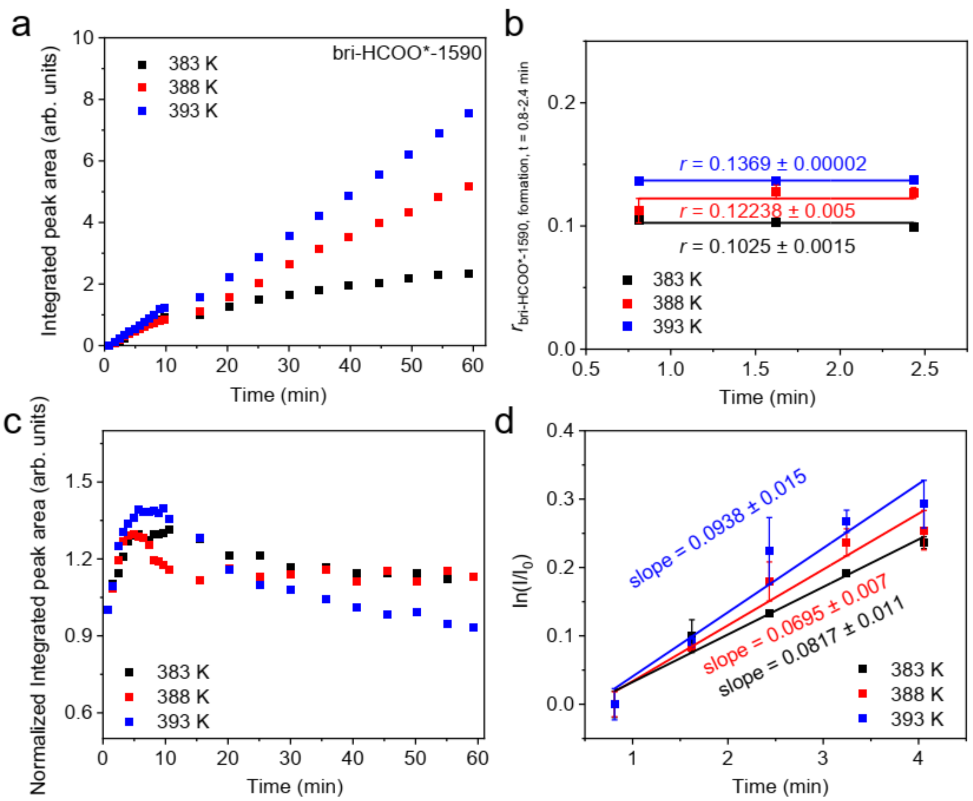

Supplementary Figure 61. a, the corresponding normalized integrated peak area and **b,** the average rate of observed bri-HCOO*-1590 species and **c** to **d,** OH* species in Supplementary Figure 60 as a function of time on Zn20Zr80 catalyst. The error bars in the figure represent the standard errors (SE) of the fitted values.

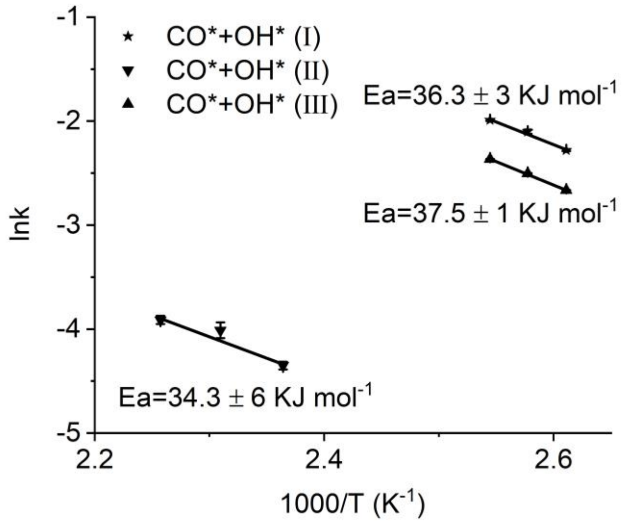

Supplementary Figure 62. Arrhenius plots of bri-HCOO* formation reaction on Zn20Zr80 derived from Supplementary Figure 59 and Supplementary Figure 61. CO+OH*(I) shows the activation energy calculated from $r_{1,app,1590}$ and CO+OH*(II) from $k_{1,app,3696}$ with bri-HCOO*-1590 prepared under 3 Mpa CO. CO+OH*(III) shows the activation energy calculated from $r_{1,app,1590}$ with bri-HCOO*-1590 prepared under 3 Mpa CO+H_2. The error bars in the figure represent the standard errors (SE) of the fitted values.

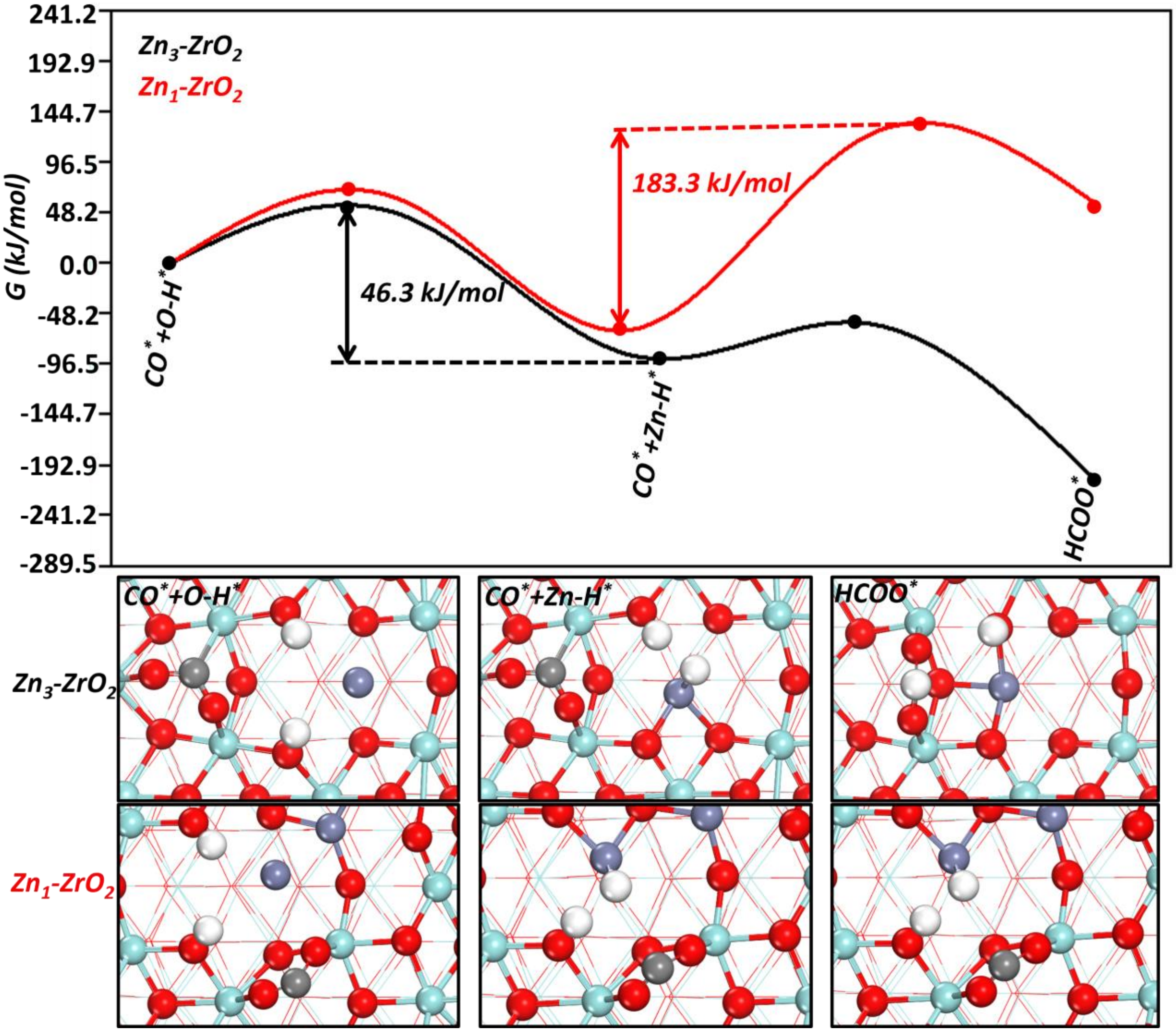

Supplementary Figure 63. Top figure: The energy profile of H diffusion and HCOO production on Zn_3-ZrO_2 and Zn_1-ZrO_2; bottom figure: the snapshots of reaction species.

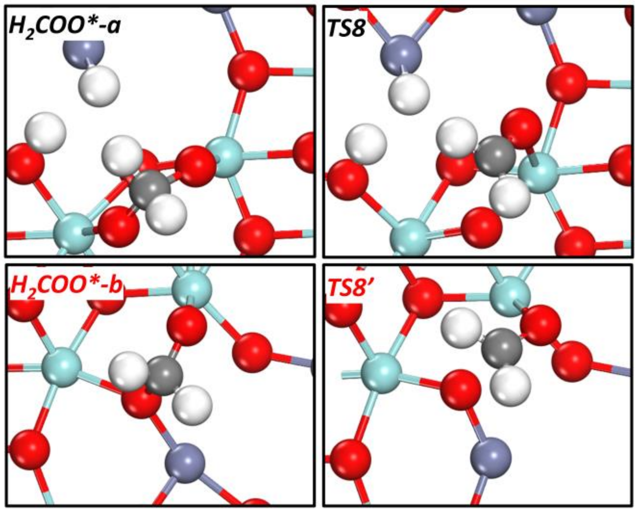

Supplementary Figure 64. The snapshots of rate-limiting steps in CO hydrogenation corresponding to Fig. 8a.

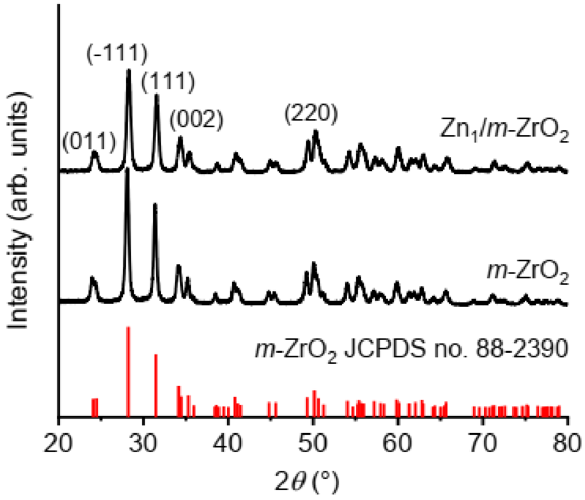

Supplementary Figure 65. XRD pattern of Zn_1/*m*-ZrO_2 and *m*-ZrO_2 support.

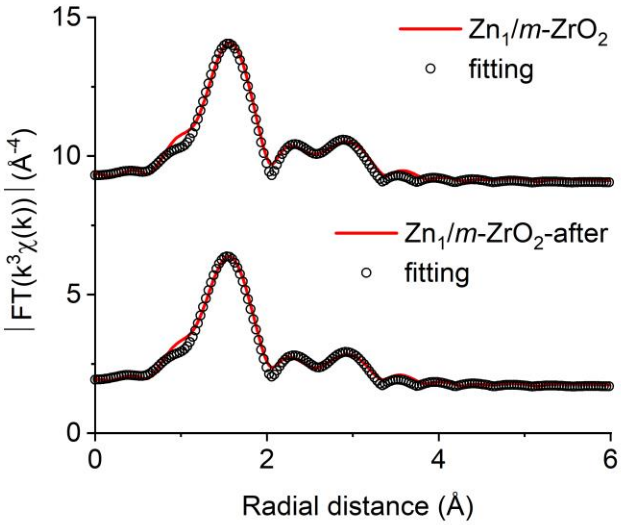

Supplementary Figure 66. Zn K-edge EXAFS fitting analyses for Zn_1/*m*-ZrO_2 before and after reaction samples in R Space.

Supplementary Table 8. EXAFS curve-fitting parameters for corresponding samples. [a]

Sample	Coordination	CN	R(Å)	$\sigma^2\times10^{-3}$(Å^2)	ΔE(eV)	R-factor
Zn foil	Zn-Zn	6	2.64	--	--	--
Zn_1/*m*-ZrO_2	Zn-O	4	2.00	8.97	0.97	0.43%
ZnO	Zn-O	4	1.97			
	Zn-O-Zn	12	3.21			

[a] CN, coordination number; R, distance between absorber and backscatter atoms; σ^2, Debye-Waller factor to account for both thermal and structural disorders; ΔE, inner potential correction; R-factor indicates the goodness of the fit.

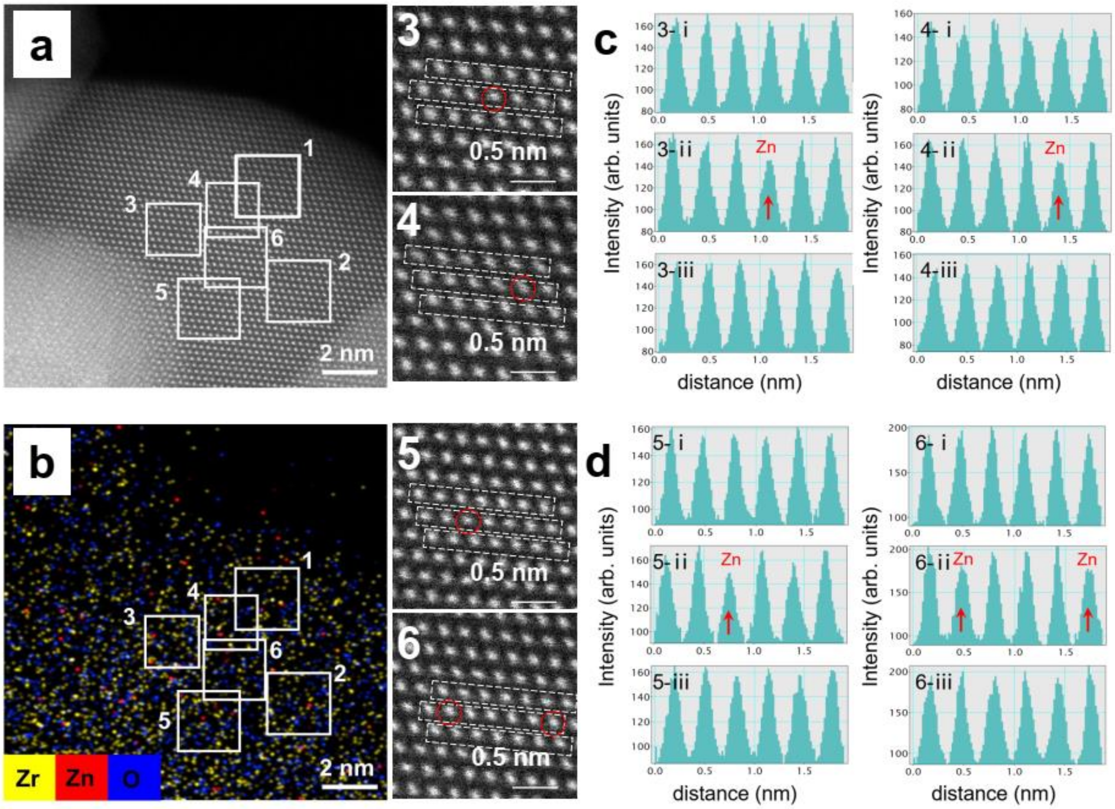

Supplementary Figure 67. a, atomically resolved STEM spectrum imaging the edge of the nanoparticle. **b,** EDS mapping images of the Zn_1/*m*-ZrO_2 catalyst. **c** to **d,** the corresponding single-pixel line profiles across the atom rows marked by rectangles in panel 3-i, 3-ii, 3-iii; 4-i, 4-ii, 4-iii; 5-i, 5-ii, 5-iii; 6-i, 6-ii, 6-iii. The dark dots and low-intensity single-pixel line profiles correspond to Zn-containing atomic columns, marked with red circles and arrows.

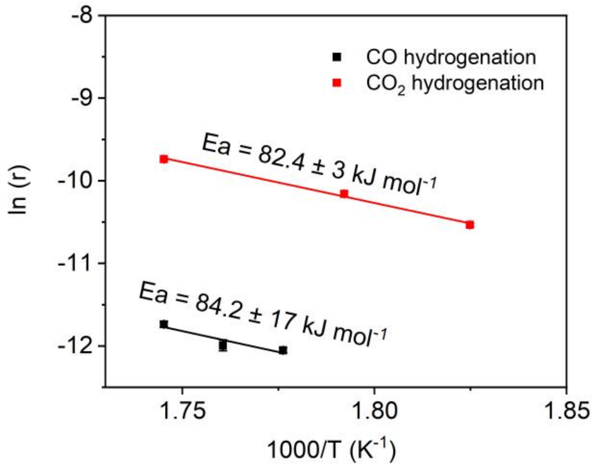

Supplementary Figure 68. Arrhenius plot of methanol formation rate derived from Fig. 9e on Zn_1/*m*-ZrO_2 in the CO and CO_2 hydrogenation to methanol reaction. The error bars in the figure represent the standard errors (SE) of the fitted values.

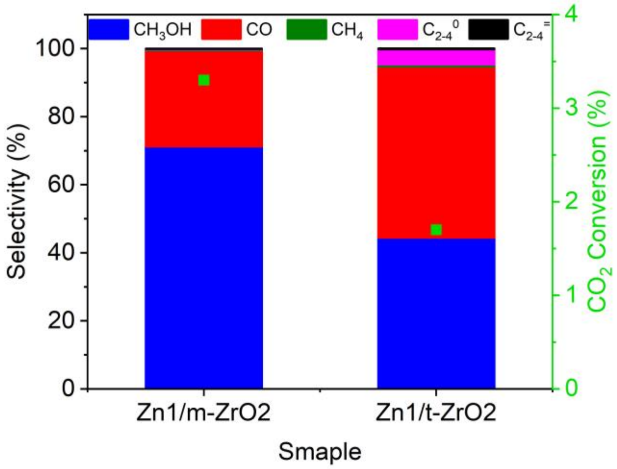

Supplementary Figure 69. CO_2 hydrogenation activity of Zn_1/*m*-ZrO_2 and Zn_1/*t*-ZrO_2 (P = 3.0 MPa; H_2: CO_2 = 3; flow rate: 30 mL min^{-1}; catalyst mass: 300 mg)

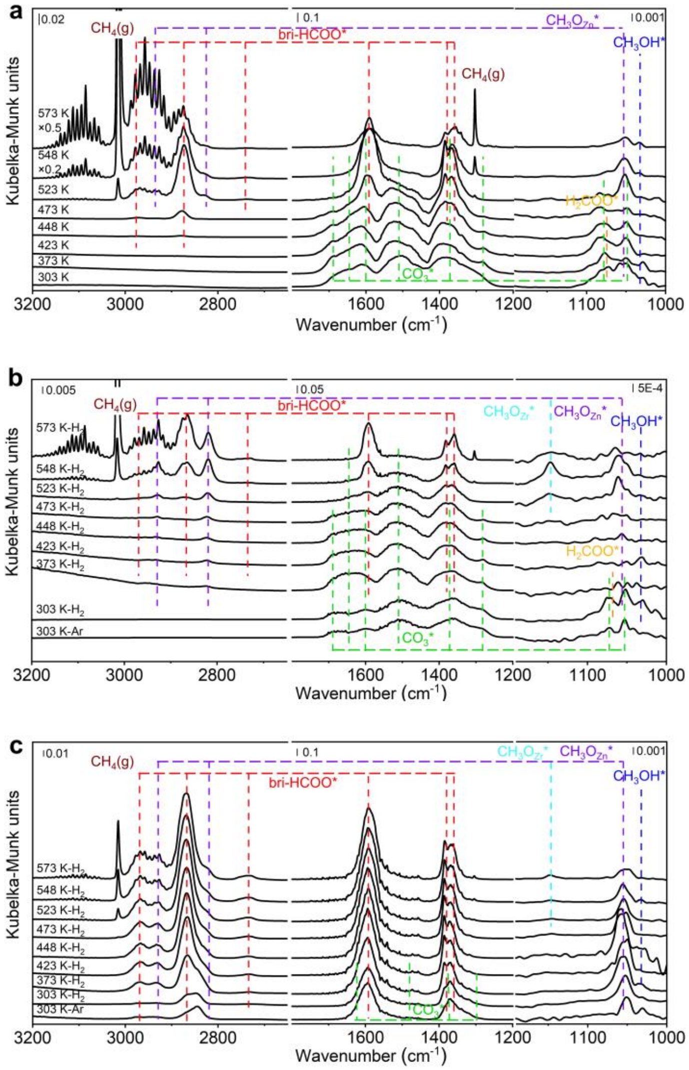

Supplementary Figure 70. Steady-state in situ DRIFTS spectra at indicated temperatures of Zn_1/*m*-ZrO_2 **a,** CO_2+H_2. **b,** CO_2→H_2. and **c**, CO_2+H_2→H_2. The processing method is consistent with Zn20Zr80. For CO_2+H_2 and CO_2→H_2 experiments the bri-HCOO* species appeared at 373 K and maximize at 573 K and the H_2COO*, CH_3O_{Zn}*, and CH_3OH* appeared significantly at 303 K and could be observed under 448 K; the CH_3O_{Zn}* and CH_3OH* species almost disappeared at 423 K (CO_2→H_2) or 473 K (CO_2+H_2), reappeared at 473 K. For CO_2+H_2→H_2 experiments, the bri-HCOO* species almost remain unchanged with temperature increased and the CH_3O_{Zn}* species could be hydrogenated above 473 K.

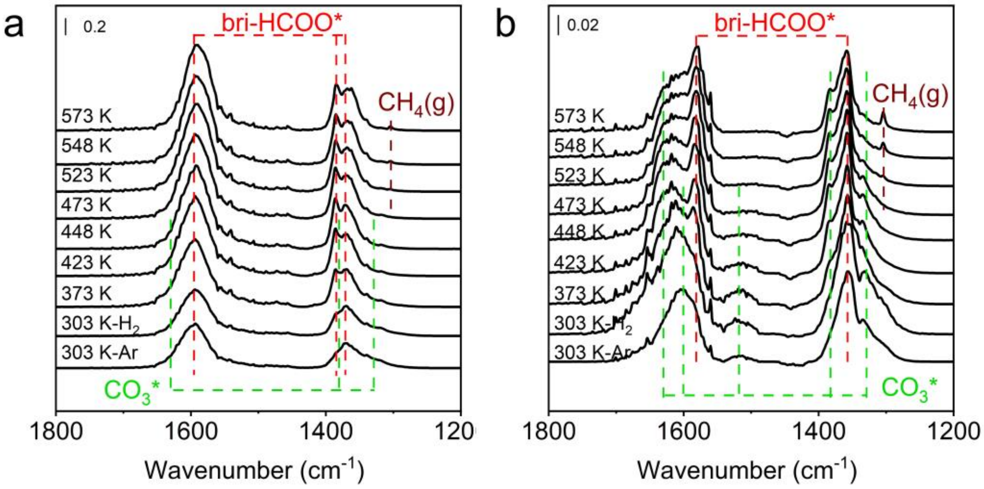

Supplementary Figure 71. In situ DRIFTS spectra of **a,** Zn_1/*m*-ZrO_2 and **b,** *m*-ZrO_2 exposed to 3 Mpa H_2 (pretreated in 3 MPa CO_2+H_2 (H_2: CO_2=3) at 573 K for 60 min, then purged in Ar) under 303 K to 573 K. The bri-HCOO* species on the surfaces of Zn_1/*m*-ZrO_2 and *m*-ZrO_2 samples exhibited similar hydrogenation reactivity that almost no hydrogenation performance at 573 K high temperature, which speculate the continuously accumulating bri-HCOO* species observed in Zn_1/*m*-ZrO_2 samples above 523 K mainly come from the generous exposed Zr-O-Zr sites on the surface.

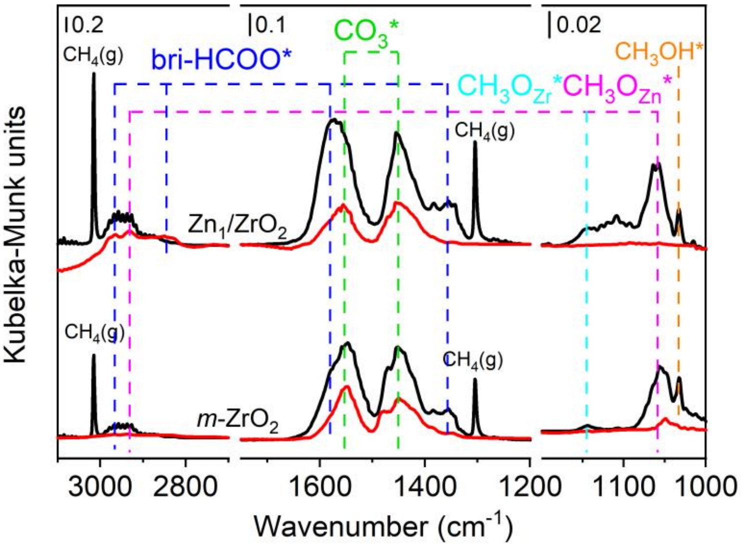

Supplementary Figure 72. In situ DRIFTS spectra of Zn_1/*m*-ZrO_2 and *m*-ZrO_2 exposed to 3 Mpa CO+H_2 (H_2: CO=2) at 573 K for 60 min (black line), then purged in Ar (red line) under 303 K.

Supplementary Table 9. Computed thermodynamic values of reactants and products in the unit of kJ mol^{-1}. E_{DFT} and E_{ZPE} represent the computed potential energy and zero-point energy, respectively. $(H\text{-}\Delta TS)_{573K}$ and $RTln(p/p^0)$ correspond to the thermodynamic corrections under reaction conditions. ΔH_{corr}^{Θ} refers to the enthalpy correction under standard conditions. G and H^{Θ} ($H=E_{DFT}+E_{ZPE}+\Delta H_{corr}^{\Theta}$) denote the calculated Gibbs free energy under the reaction condition and the enthalpy under the standard condition, respectively. The enthalpy changes and entropies for CO, CO_2, H_2, and H_2O under reaction and standard conditions, as well as for CH_3OH under standard conditions, are obtained from the NIST-JANAF Thermochemical Tables. The enthalpy change and entropy of CH_3OH under the reaction condition are calculated from DFT. All energies are in the unit of kJ mol^{-1}.

	E_{DFT}	E_{ZPE}	Reaction condition (573 K, 3 MPa, H_2: CO_2 = 3)			Standard condition	
			$(H\text{-}\Delta TS)_{573K}$	$RTLn(p/p^{\Theta})$	G	ΔH_{corr}^{Θ}	H^{Θ}
CO_2 (g)	-2217.23	29.91	-117.71	9.55	-2295.38	9.65	-2177.67
H_2O (g)	-1372.02	54.03	-101.31	-10.61	-1429.91	9.65	-1308.34
H_2 (g)	-654.16	26.05	-69.47	14.47	-683.11	8.68	-619.43
CH_3OH (g)	-2914.81	131.22	-130.25	-10.61	-2924.46	10.61	-2772.98

Using these data, the $\Delta G= G(CH_3OH(g))+G(H_2O(g))-3*G(H_2(g))-G(CO_2(g))$ was calculated as -9.9 kJ mol^{-1} at the reaction condition, close to the equilibrium, while the $\Delta H^{\Theta}= H^{\Theta}(CH_3OH(g))+ H^{\Theta}(H_2O(g))-3* H^{\Theta}(H_2(g))- H^{\Theta}(CO_2(g))$ was calculated as -45.3 kJ mol-1, close to the experimental value (-49.5 kJ mol^{-1}).

Supplementary References

1. Deroubaix, G. & Marcus, P. X-ray photoelectron spectroscopy analysis of copper and zinc oxides and sulphides. *Surf. Interface Anal.* **18**, 39–46 (1992).
2. Iwai, H., Umeki, T., Yokomatsu, M. & Egawa, C. Methanol partial oxidation on Cu–Zn thin films grown on Ni(100) surface. *Surf. Sci.* **602**, 2541–2546 (2008).
3. Wang, Y. et al. Exploring the ternary interactions in Cu–ZnO–ZrO_2 catalysts for efficient CO_2 hydrogenation to methanol. *Nat Commun* **10**, 1166 (2019).
4. Lin, L., Wang, G. & Zhao, F. CO_2 hydrogenation to methanol on ZnO/ZrO_2 catalysts: Effects of zirconia phase. *ChemistrySelect.* **6**, 2119–2125 (2021).
5. Li, H. et al. Ni/SBA-15 catalysts for CO methanation: Effects of V, Ce, and Zr promoters. *RSC Adv.* **5**, 96504–96517 (2015).
6. Shido, T. & Iwasawa, Y. Reactant-promoted reaction mechanism for water-gas shift reaction on ZnO, as the genesis of surface catalysis. *J. Catal.* **129**, 343–355 (1991).
7. Xu, C. & Goodman, D. W. Adsorption and reaction of formic acid on the Mo(110) and O/Mo(110) surfaces. *J. Phys. Chem.* **100**, 1753–1760 (1996).
8. Avery, N. R. Adsorption of formic acid on clean and oxygen covered Pt(111). *Appl. Surf. Sci.* **11–12**, 774–783 (1982).
9. Durand, J. P., Senanayake, S. D., Suib, S. L. & Mullins, D. R. Reaction of formic acid over amorphous manganese oxide catalytic systems: An in-situ study. *J. Phys. Chem. C.* **114**, 20000–20006 (2010).
10. Bossola, F. et al. Electron-poor copper nanoparticles over amorphous zirconia-silica as all-in-one catalytic sites for the methanol steam reforming. *Appl. Catal., B.* **258**, 118016 (2019).
11. Guglielminotti, E. Infrared study of syngas adsorption on zirconia. *Langmuir.* **6**, 1455–1460 (1990).
12. Fujita, S., Usui, M., Ito, H. & Takezawa, N. Mechanisms of methanol synthesis from carbon dioxide and from carbon monoxide at atmospheric pressure over Cu/ZnO. *J. Catal.* **157**, 403–413 (1995).
13. Wang, X., Zhang, H. & Li, W. In situ IR studies on the mechanism of methanol synthesis from CO/H_2 and CO_2/H_2 over Cu-ZnO-Al_2O_3 catalyst. *Korean J. Chem. Eng.* **27**, 1093–1098 (2010).
14. Arandia, A. et al. Effect of atomic layer deposited zinc promoter on the activity of copper-on-zirconia catalysts in the hydrogenation of carbon dioxide to methanol. *Appl. Catal., B.* **321**, 122046 (2023).
15. Idriss, H. et al. Characterization of dioxymethylene species over Cu-Zn catalysts. *J. Mol. Catal.* **42**, 205–213 (1987).
16. Zhang, Y. et al. Interaction of formaldehyde with the rutile TiO_2(110) surface: A combined experimental and theoretical study. *J. Phys. Chem. C.* **120**, 12626–12636 (2016).
17. Takeyasu, K. et al. Hydrogenation of formate species using atomic hydrogen on a Cu(111) model catalyst. *J. Am. Chem. Soc.* **144**, 12158–12166 (2022).
18. Cao, Q., Berski, S., Latajka, Z., Räsänen, M. & Khriachtchev, L. Reaction of atomic hydrogen with formic acid. *Phys. Chem. Chem. Phys.* **16**, 5993 (2014).
19. Noei, H., Wöll, C., Muhler, M. & Wang, Y. Activation of carbon dioxide on ZnO nanoparticles studied by vibrational spectroscopy. *J. Phys. Chem. C.* **115**, 908–914 (2011).
20. Kouva, S. et al. Water and carbon oxides on monoclinic zirconia: Experimental and computational insights. *Phys. Chem. Chem. Phys.* **16**, 20650–20664 (2014).
21. Bolis, V., Magnacca, G., Cerrato, G. & Morterra, C. Microcalorimetric and IR-spectroscopic

study of the room temperature adsorption of CO_2 on pure and sulphated *t*-ZrO_2. *Thermochim Acta.* **379**, 147–161 (2001).
22. Hill, I. M., Hanspal, S., Young, Z. D. & Davis, R. J. DRIFTS of probe molecules adsorbed on magnesia, zirconia, and hydroxyapatite catalysts. *J. Phys. Chem. C.* **119**, 9186–9197 (2015).
23. Na, B. K., Walters, A. B. & Vannice, M. A. Studies of gas adsorption on ZnO using ESR, FTIR spectroscopy, and MHE (microwave hall effect) measurements. *J. Catal.* **140**, 585–600 (1993).
24. Fu, C. et al. Site sensitivity of interfacial charge transfer and photocatalytic efficiency in photocatalysis: Methanol oxidation on anatase TiO_2 nanocrystals. *Angew. Chem. Int. Ed.* **60**, 6160–6169 (2021).
25. de Souza, P. M. et al. Effect of zirconia morphology on hydrodeoxygenation of phenol over Pd/ZrO_2. *ACS Catal.* **5**, 7385–7398 (2015).
26. Kouva, S., Honkala, K., Lefferts, L. & Kanervo, J. Review: monoclinic zirconia, its surface sites and their interaction with carbon monoxide. *Catal. Sci. Technol.* **5**, 3473–3490 (2015).
27. Guo, J., Hou, Z., Gao, J. & Zheng, X. DRIFTS study on adsorption and activation of CH_4 and CO_2 over Ni/SiO_2 catalyst with various Ni particle sizes. *Chinese J. Catal.* **28**, 22–26 (2007).
28. Kenzhin, R. M., Volodin, A. M. & Bedilo, A. F. Reactivity of O_2^- radical anions on hydrated ZrO_2 surface. *Res. Chem. Intermed* **39**, 1789–1797 (2013).
29. Wright, S. & Barklie, R. C. EPR characterization of defects in monoclinic powders of ZrO_2 and HfO_2. *Mater. Sci. Semicond. Process* **9**, 892–896 (2006).
30. Zhao, M. et al. Electron spin resonance (ESR) studies on the interaction of oxygen with semiconductors under photoirradiation. *Chem. Pharm. Bull* **16**, 1240–1243 (1968).
31. Song, L., Cao, X. & Li, L. Engineering stable surface oxygen vacancies on ZrO_2 by hydrogen-etching technology: An efficient support of gold catalysts for water-gas shift reaction. *ACS Appl. Mater. Interfaces.* **10**, 31249–31259 (2018).
32. Liu, H., Feng, L., Zhang, X. & Xue, Q. ESR characterization of ZrO_2 nanopowder. *J. Phys. Chem.* **99**, 332–334 (1995).
33. Zhang, L. et al. Activation and surface reactions of CO and H_2 on ZnO powders and nanoplates under CO hydrogenation reaction conditions. *J. Energy Chem.* **50**, 351–357 (2020).
34. Zhao, Q., Wang, X. & Cai, T. The study of surface properties of ZrO_2. *Appl. Surf. Sci.* **225**, 7–13 (2004).
35. Pinheiro Araújo, T. et al. Flame-made ternary Pd-In_2O_3-ZrO_2 catalyst with enhanced oxygen vacancy generation for CO_2 hydrogenation to methanol. *Nat Commun* **13**, 5610 (2022).
36. Zhang, M. et al. Controlled formation of native defects in ultrapure ZnO for the assignment of green emissions to oxygen vacancies. *J. Phys. Chem. C.* **124**, 12696–12704 (2020).
37. Zhang, M. et al. Defect-related multicolor emissions in ZnO smoke: from violet, over green to yellow. *Nanoscale* **11**, 5102–5115 (2019).
